# Growth Light Environment Changes the Sensitivity of Photosystem I Photoinhibition Depending on Common Wheat Cultivars

**DOI:** 10.3389/fpls.2019.00686

**Published:** 2019-06-04

**Authors:** Daisuke Takagi, Hiroaki Ihara, Shigeo Takumi, Chikahiro Miyake

**Affiliations:** ^1^ Department of Biological and Environmental Science, Graduate School of Agricultural Science, Kobe University, Kobe, Japan; ^2^ Core Research for Environmental Science and Technology, Japan Science and Technology Agency, Tokyo, Japan

**Keywords:** photosystem I, photoinhibition, reactive oxygen species, light acclimation, wheat (*Triticum aestivum* L.)

## Abstract

Light is an important factor for determining photosynthetic performance in land plants. At high light intensity, land plants develop photosynthetic activity by increasing electron sinks, such as the Calvin cycle and photorespiration and photoprotective mechanisms in photosystem II (PSII), to effectively utilize light and protect them from photoinhibition. In addition to PSII, photosystem I (PSI) has a risk of undergoing photoinhibition under high light intensity because of the reactive oxygen species (ROS) produced within PSI. However, the acclimation response has hardly been evaluated in the relationship of PSI photoprotection to growth light. In this study, we studied the effect of growth light intensity on the photoprotective mechanisms in PSI using six wheat cultivars. To evaluate the susceptibility of PSI to its photoinhibition, we used the repetitive short-pulse (rSP) illumination method to cause O_2_-dependent PSI photoinhibition. We found that PSI photoinhibition induced by rSP illumination was much more alleviated in wheat cultivars grown under high-light conditions compared to those grown under low-light conditions. Here, we observed that wheat plant grown under high-light conditions lowered the susceptibility of PSI to its photoinhibition compared to those grown under low-light conditions. Furthermore, the acclimation response toward PSI photoinhibition was significantly different among the studied wheat cultivars, although the quantum yields both of PSII and PSI were increased by high-light acclimation in all wheat cultivars as reported previously. Interestingly, we observed that total chlorophyll content in leaves correlated with the susceptibility of PSI to its photoinhibition. On the basis of these results, we suggest that high-light acclimation induces protection mechanisms against PSI photoinhibition in land plants, and the increase in the leaf chlorophyll content relates to the susceptibility of PSI photoinhibition in wheat plants.

## Introduction

Land plants grow by photosynthesizing at the sites where they germinate and perform their life cycles even under an unfavorable growth environment. The growth environment greatly affects photosynthetic activity in land plants. For example, a change in temperature affects the carbon fixation rate by influencing the activities or activation states of enzymes involved in the Calvin cycle and other related metabolic processes in the mitochondria and cytoplasm (Atkin and Tjoelker, 2003; Makino and Sage, 2007; Yamori et al., 2014; Noguchi et al., 2015). Moreover, a balance between the rates of carbon reduction in the Calvin cycle and carbon oxidation in photorespiration fluctuates depending on the temperature (Sage et al., 2008). The water status in the soil culture is also important; drought induces stomatal closure and suppresses photosynthesis by limiting the CO_2_ supply in leaves (Zivcak et al., 2013). Furthermore, a fluctuating light environment disturbs the redox state in thylakoid membranes and stimulates the production of reactive oxygen species (ROS) during photosynthesis (Tikkanen et al., 2010, 2012; Alter et al., 2012; Suorsa et al., 2012; Kono et al., 2014).

To accomplish their life-cycles, land plants possess an ability to maximize photosynthesis under their growth conditions. They can acclimate to fluctuating environmental conditions by optimizing their photosynthetic ability (Walters, 2005; Ensminger et al., 2006; Yamori et al., 2014; Schumann et al., 2017). Acclimation in land plants is comprised of many macro-responses, such as a change in leaf morphology, and micro-responses, such as a change in gene expression or protein composition (De la Torre and Burkey, 1990; Hüner et al., 1998, 2014; Bailey et al., 2001; Savitch et al., 2001; Oguchi et al., 2003; Walters et al., 2003; Dahal et al., 2012; Dumlao et al., 2012; Cohu et al., 2014). Light intensity and quality are two of the most important factors for determining leaf protein composition and morphology (Suorsa et al., 2015; Bielczynski et al., 2016; Schumann et al., 2017). In high-light (HL)-acclimated plants, an increase in leaf thickness, leaf nitrogen content, and photosynthetic protein [Rubisco, Photosystem II (PSII), Cytochrome *b_6_f*, etc.] is observed compared to the plants grown under low-light (LL) conditions (De la Torre and Burkey, 1990; Bailey et al., 2001; Oguchi et al., 2003; Walters et al., 2003; Dietz, 2015; Bielczynski et al., 2016; Schumann et al., 2017). Because of these acclimation responses, the photosynthetic rate on leaf area basis increases in acclimated plants compared to non-acclimated plants (Oguchi et al., 2003; Dahal et al., 2012; Schumann et al., 2017). In addition to the photosynthetic rate, the photoprotective mechanisms in PSII are also strengthened by HL acclimation. For protecting PSII from photoinhibition, various photoprotective mechanisms, such as non-photochemical quenching (NPQ), state-transition, and PSII repair-cycle, have been proposed (Müller et al., 2001; Nishiyama et al., 2011; Goldschmidt-Clermont and Roberto, 2015; Liu and Last, 2017). These mechanisms respond to the growth light environment, allowing the land plants to modulate their photoprotective capacity (Miyata et al., 2015; Ware et al., 2015; Schumann et al., 2017). As a result, the land plants grown under HL conditions showed higher tolerance against PSII photoinhibition compared to the plants grown under LL conditions (Gray et al., 1996, 1997; Pocock et al., 2001; Dietz, 2015; Miyata et al., 2015).

As described above, several reports have focused on differences in photosynthetic activity, particularly the differences in electron sink capacity and PSII photoprotective mechanisms of plants. However, the effect of the photoprotective mechanisms of PSI during growth light acclimation has rarely been discussed (Sonoike et al., 1995a,b; Sonoike, 1996a, 1998; Ivanov et al., 1998). PSI causes photoinhibition by the ROS produced within the thylakoid membranes when the photosynthetic electron transport chain is highly reduced (Sonoike and Terashima, 1994; Terashima et al., 1994; Sonoike, 1995, 1996a, 1996b, 1999, 2011; Sonoike et al., 1995a,b; Sejima et al., 2014; Takagi et al., 2016a). For protecting PSI from its photoinhibition, the P700 reaction center chlorophyll (P700) oxidation system is primarily important (Sejima et al., 2014; Shimakawa et al., 2016, 2017, 2019; Takagi et al., 2016b, 2017a,b). The P700 oxidation system consists of the donor side and the acceptor side photosynthetic electron transport regulation mechanisms. The donor side-dependent P700 oxidation system is further separated into the ΔpH-dependent and ΔpH-independent reactions termed as the reduction-induced suppression of photosynthetic electron flow (RISE) reactions (Shaku et al., 2015; Shimakawa et al., 2017; Takagi et al., 2017a,b). At the acceptor side of PSI, photorespiration and flavodiiron proteins contribute to P700 oxidation by stimulating electron outflow from PSI (Shimakawa et al., 2016, 2017; Takagi et al., 2016b, 2017a). The increase in oxidized P700 reduces the risk of over-reduction state within PSI due to the relative decrease in the ground state of P700 and is capable of reducing ROS production within the thylakoid membranes (Sejima et al., 2014; Takagi et al., 2016a, 2017a). Importantly, P700 oxidation is greatly stimulated at the sites where CO_2_ fixation is limited (Miyake et al., 2005; Takagi et al., 2016b). Several studies have revealed that the P700 oxidation systems contribute to P700 oxidation to prevent the ROS-induced PSI photoinhibition (Shaku et al., 2015; Shimakawa et al., 2016, 2017; Takagi et al., 2016b, 2017b). Therefore, the P700 oxidation systems are fundamental protective mechanisms to escape PSI photoinhibition in photosynthetic organisms.

In addition to the P700 oxidation system, PSI photoinhibition can be modulated independently of the photosynthetic electron transport regulation. Takagi et al. (2017a) reported that angiosperms show a different susceptibility of PSI to photoinhibition, which is independent of P700 oxidation. However, the molecular mechanisms of this difference have remained unknown. Because the difference in the susceptibility of PSI to photoinhibition was observed among the angiosperms growing at different sites in the field, it is ambiguous to predict whether this difference among angiosperms is physiologically controllable within the same plant species.

In the present study, we characterized the effect of growth light conditions on the susceptibility of PSI to its photoinhibition by using six wheat cultivars. To determine the effect of the growth light conditions on the susceptibility of PSI to its photoinhibition in intact leaves, we used repetitive short-pulse (rSP) illumination for wheat cultivars grown under LL (50–60 μmol photons m^−2^ s^−1^) and HL (700–800 μmol photons m^−2^ s^−1^) conditions. Subsequently, we found that the HL-acclimated wheat plants showed lower susceptibility to PSI photoinhibition compared to the LL-acclimated wheat cultivars. Furthermore, we observed cultivar differences in the susceptibility of PSI photoinhibition to growth light conditions, even though the quantum yield of photosystem (PS) II and PSI showed acclimation responses to growth light conditions among all wheat cultivars. Here, we discussed a new insight for protecting PSI from its photoinhibition.

## Materials and Methods

### Plant Materials and Growth Conditions

In this study, we used six cultivars of wheat (*Triticum aestivum* L.), including three spring-type cultivars—“Chinese Spring” (CS), “Bobwhite” (Bob), and “Haruyokoi” (Haru)—and three winter-type cultivars—“Mironovskaya 808” (M808), “Norin 61” (N61), and “Akadaruma” (Aka). Seeds were imbibed with wet cotton at 4°C for 3 days to promote synchronized germination. The imbibed seeds were grown in a mixture of soil (Metro-Mix 350; Sun Gro Horticulture, Bellevue, WA, USA) and vermiculite (Konan, Osaka, Japan) in pots (7.5 cm × 7.5 cm in width and 6 cm in depth). The plants were placed in an environmentally controlled chamber with a photoperiod of 14 h light (25°C) and 10 h dark (20°C). The light intensity was 700–800 μmol photons m^−2^ s^−1^ (high-light condition) or 50–60 μmol photons m^−2^ s^−1^ (low-light condition). The seedlings were watered every second day with 0.1% Hyponex solution (N:P:K = 5:10:5, Hyponex, Osaka, Japan). For the analysis, we used the fully expanded mature leaves of the plants grown for at least 6 weeks.

### Measurement of Chlorophyll Content in Wheat Leaves

The leaf chlorophyll content and chlorophyll *a*/*b* ratio were determined as reported previously (Takagi et al., 2017b). In brief, leaf segments were incubated in *N, N*-dimethylformamide at 4°C overnight, followed by the extraction of chlorophylls from leaves. The absorbances of the aliquots were measured at 750, 663.8, and 646.8 nm to calculate the chlorophyll content (Porra et al., 1989).

### Measurement of Chlorophyll Fluorescence and P700^+^

Chlorophyll fluorescence and P700^+^ were simultaneously measured with the Dual-PAM-100 system (Heintz Walz GmbH, Effeltrich, Germany) equipped with a 3010 Dual gas exchange chamber (Heintz Walz GmbH, Effeltrich, Germany). The atmospheric gas (40 Pa CO_2_/21 kPa O_2_) and the gas with the indicated mixture of pure O_2_ and CO_2_, as prepared by mixing 20.1% (v/v) O_2_ in 79.9% (v/v) N_2_, 1% (v/v) CO_2_ in 99% N_2_, and pure N_2_ gas using a mass-flow controller (Kofloc model 1203; Kojima Instrument Co.), were used in this study. The gases were saturated with water vapor at 13.5 ± 0.1°C, and the leaf temperature was controlled at 25°C. The chlorophyll fluorescence parameters were calculated as follows (Baker, 2008): maximum quantum efficiency of PSII photochemistry, Fv/Fm = (Fm − Fo)/Fm; quantum yield of photochemical energy conversion in PSII, Y(II) = (Fm′ − Fs)/Fm′, where Fo is the minimum fluorescence yield, Fm is the maximum fluorescence yield, and Fs is the steady-state fluorescence yield; non-photochemical quenching (NPQ) = (Fm – Fm′)/Fm′. To determine Fo and Fm, measuring light (0.1 μmol photons m^−2^ s^−1^) and saturating pulse (20,000 μmol photons m^−2^ s^−1^, 300 ms) were applied. The oxidation-reduction state of P700^+^ was determined according to the methods of Klughammer and Schreiber (1994) as follows: quantum yield of photochemical energy in PSI, Y(I) = (Pm′ − P)/Pm; quantum yield of non-photochemical quenching due to acceptor side limitation, Y(NA) = (Pm − Pm′)/Pm; quantum yield of non-photochemical quenching due to donor side limitation, Y(ND) = P/Pm. The maximum oxidation level of P700 (Pm) was obtained from a saturating pulse under far-red light, reflecting the maximum amount of photooxidized P700. The parameter P reflects the steady-state oxidation level of P700, and Pm′ was obtained from the saturating pulse at a steady state. To obtain the maximized Pm′ value, Pm′ value was determined by extrapolation of the slow signal decline of 700^+^ (from 5 to 35 ms after the beginning of pulse illumination) to time zero according to Klughammer and Schreiber (1994). The zero level of P700^+^ after one pulse illumination was determined at the steady-state level of P700 in the dark for 1 s. Actinic light (AL), a mixture of red and blue lights, was used to measure the photosynthetic parameters. Light curve analysis was conducted at light intensities 48, 186, 289, 447, 788, 1,134, 1,625, and 2,439 μmol photons m^−2^ s^−1^ and the photosynthetic parameters were obtained at the steady-state condition.

### Repetitive Short-Pulse Illumination

rSP illumination was applied to the dark-adapted wheat leaves, as described previously (Sejima et al., 2014). Before this measurement, wheat plants were adapted in the dark at least for 1 h. A saturating pulse (20,000 μmol photons m^−2^ s^−1^, 300 ms) was used to illuminate the plants every 10 s in the absence of AL for 1 h under the ambient conditions (40 Pa CO_2_, 21 kPa O_2_) at 25°C. After rSP illumination, the change in Fv/Fm and Pm was determined as described in the figure legends. The kinetics of the oxidized P700 was determined at the beginning of rSP illumination, averaging all pulse kinetics in the first 1 min of rSP illumination (Takagi et al., 2017a). The P700^+^ signal was normalized using the Pm value before rSP illumination, and the relative changes in P700^+^ during one pulse illumination were shown.

### Statistical Analysis

All measurement data were expressed as mean value ± SD of at least three independent analyses. For the detection of differences among the studied wheat cultivars, we used the one-way analysis of variance (ANOVA), Student’s *t*-test, Tukey-Kramer honestly significant difference (HSD) test, and linear and nonlinear regression analysis. All statistical analyses were performed using Microsoft Excel 2010 (Microsoft) and Origin Pro 2019 (LightStone Corp.).

## Results

### High-Light Acclimation Increased the Quantum Yield of Both Photosystem II and Photosystem I in All Wheat Cultivars

**Figure 1:**
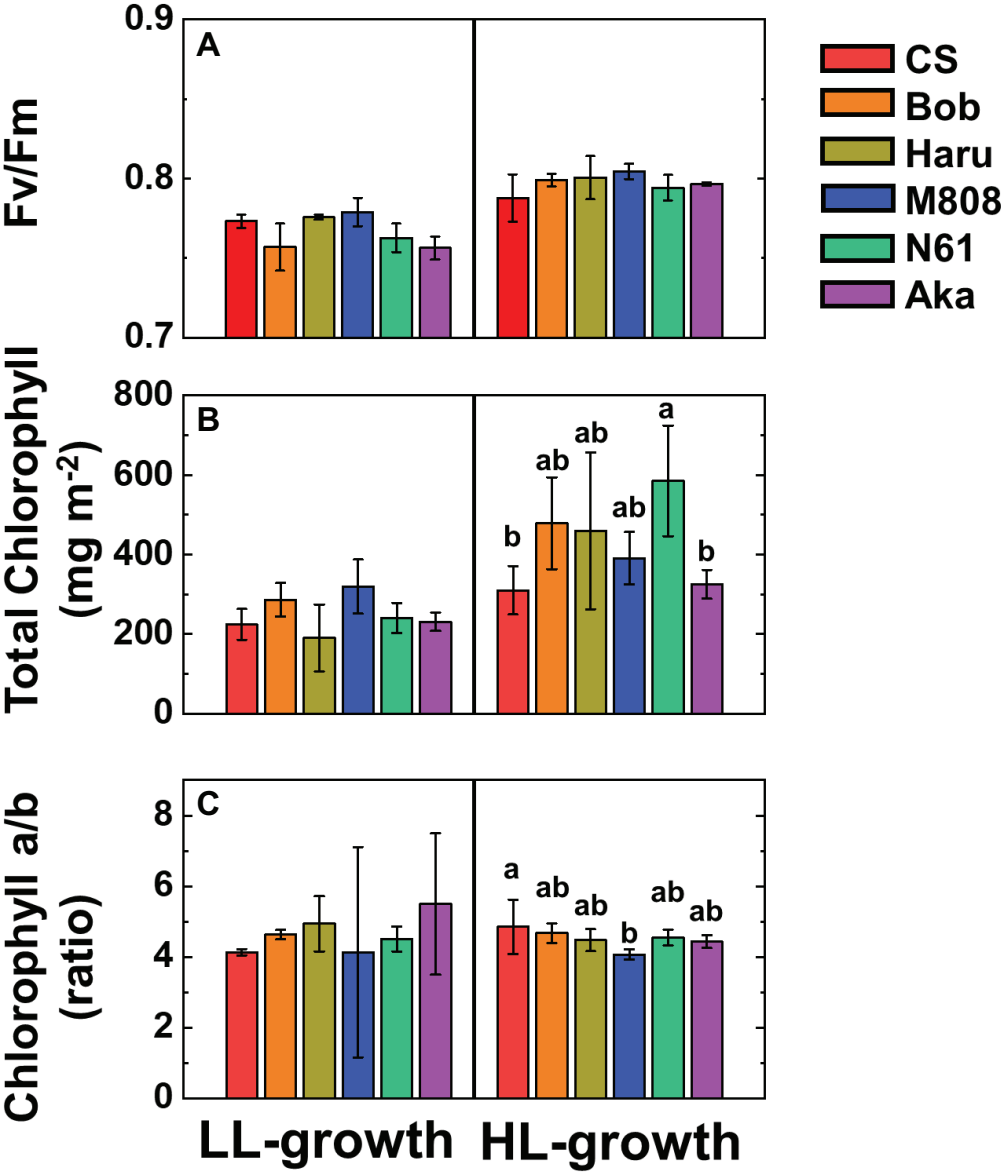
The effect of growth light conditions on the maximum quantum yield of PSII (Fv/Fm) **(A)**, total chlorophyll content on leaf area basis **(B)**, and chlorophyll *a*/*b* ratio **(C)**. Data are expressed as mean ± SD of three to five independent experiments. Different letters above the bars indicate significant differences among wheat cultivars (ANOVA and Tukey-Kramer HSD test, *p* < 0.05). Red bars indicate the results of CS, orange bars indicate the results of Bob, dark yellow bars indicate the results of Haru, blue bars indicate the results of M808, green bars indicate the results of N61, and purple bars indicate the results of Aka.

First, we studied the light response of the quantum yield of both PSII and PSI in six wheat cultivars grown under the HL and LL conditions (see Materials and Methods). In this study, we used three spring-type (CS, Bob, and Haru) and three winter-type (M808, N61, Aka) wheat cultivars. The maximum quantum yield of PSII (Fv/Fm) showed a similar value between the HL- and LL-grown wheat cultivars. Furthermore, the Fv/Fm was similar among the wheat cultivars grown under each growth light condition (Figure 1A). The total chlorophyll content was similar among the wheat cultivars grown under LL conditions (Figure 1B). In contrast, the HL-grown wheat cultivars showed significant differences, with N61 showing the highest chlorophyll content on the leaf area basis. In addition, compared to the LL-grown wheat cultivars, the HL-grown wheat cultivars showed an increase in the total chlorophyll content on the leaf area basis. This is a major HL acclimation response reported previously (De la Torre and Burkey, 1990; Hüner et al., 1998; Bailey et al., 2001; Savitch et al., 2001; Oguchi et al., 2003; Walters et al., 2003; Dahal et al., 2012; Schumann et al., 2017). The chlorophyll *a*/*b* ratio was not significantly different among the wheat cultivars grown under LL conditions (Figure 1C), whereas the HL-grown wheat cultivars showed differences in their chlorophyll *a*/*b* ratios (Figure 1C).

**Figure 2:**
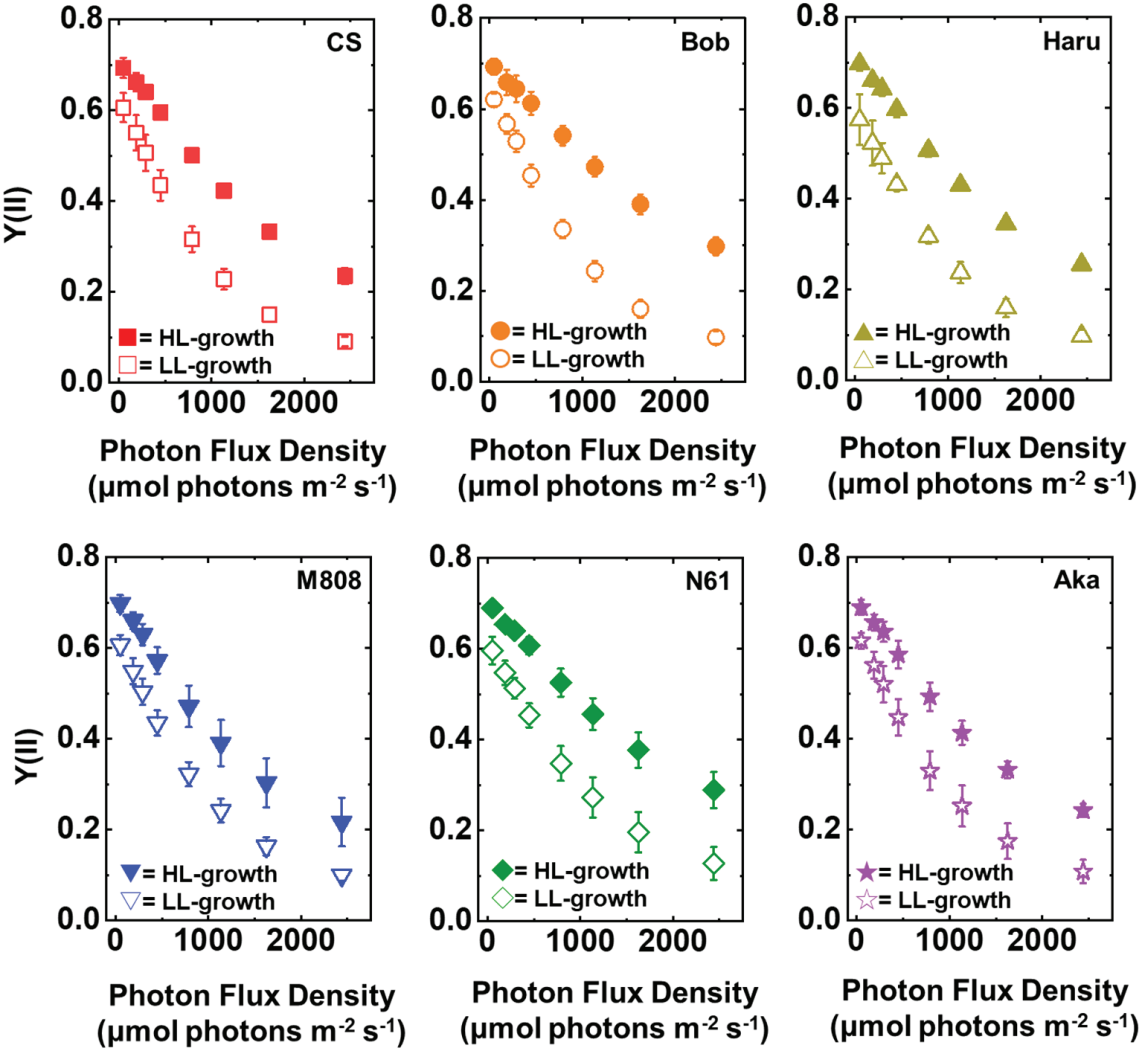
The response of Y(II) to light intensity in six wheat cultivars grown under LL (open symbol) and HL (closed symbol) conditions. The measurement was conducted under atmospheric conditions (21 kPa O_2_, 40 Pa CO_2_) at 25°C. Data were expressed as mean ± SD of three to five independent experiments. Red squares indicate the results of CS, orange circles indicate the results of Bob, dark yellow triangles indicate the results of Haru, blue inverted-triangles indicate the results of M808, green diamonds indicate the results of N61, and purple stars indicate the results of Aka.

The quantum yield of PSII [Y(II)] was compared among the six wheat cultivars grown under both LL and HL conditions (Figure 2). Under LL growth conditions, Y(II) was decreased in accordance with the increase in illuminated light intensity in all wheat cultivars, showing no significant differences among them (Figure 2, Supplementary Figure S1A). Compared to LL conditions, HL conditions increased Y(II) toward the illuminated light intensity in all wheat cultivars (Figure 2). However, no significant difference was observed among the wheat cultivars grown under HL conditions (Supplementary Figure S1B).

**Figure 3:**
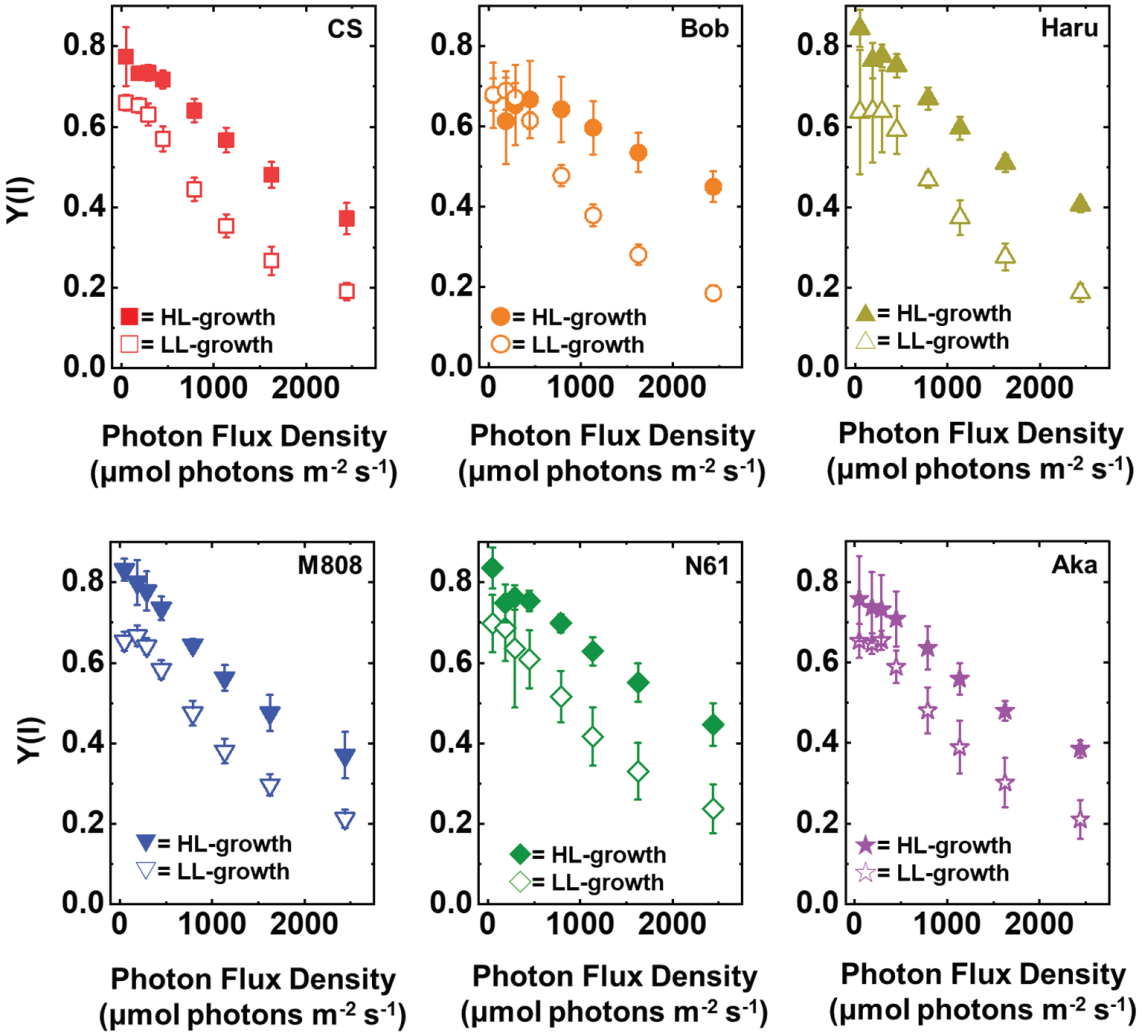
The response of Y(I) to light intensity in six wheat cultivars grown under LL (open symbol) and HL (closed symbol) conditions. The measurement was conducted under atmospheric conditions (21 kPa O_2_, 40 Pa CO_2_) at 25°C. Data were expressed as mean ± SD of three to five independent experiments. Red squares indicate the results of CS, orange circles indicate the results of Bob, dark yellow triangles indicate the results of Haru, blue inverted-triangles indicate the results of M808, green diamonds indicate the results of N61, and purple stars indicate the results of Aka.

In concert with the result of Y(II), Y(I) toward the increase in illuminated light intensity was higher in the HL-grown wheat cultivars than that in the LL-grown wheat cultivars (Figure 3). Similar to Y(II), no significant cultivar difference of Y(I) was detected among the wheat cultivars grown under both growth light conditions (Supplementary Figures S1C,D). These acclimation responses of the quantum yield of both PSII and PSI were similar to that of the previous report (Miyake et al., 2005). These results indicated that all wheat cultivars possess a capacity to change the quantum yield of both photosystems under HL conditions and increased the quantum yield of both PSII and PSI by acclimating the HL growth conditions compared to the LL growth conditions.

### High-Light-Grown Wheat Plants Showed Lower P700^+^ Under Illumination Than Low-Light-Grown Wheat Plants Although the Acceptor Side of Electron Transport Limitation in Photosystem I Was Similar Between Low-Light- and High-Light-Grown Wheat Plants

**Figure 4:**
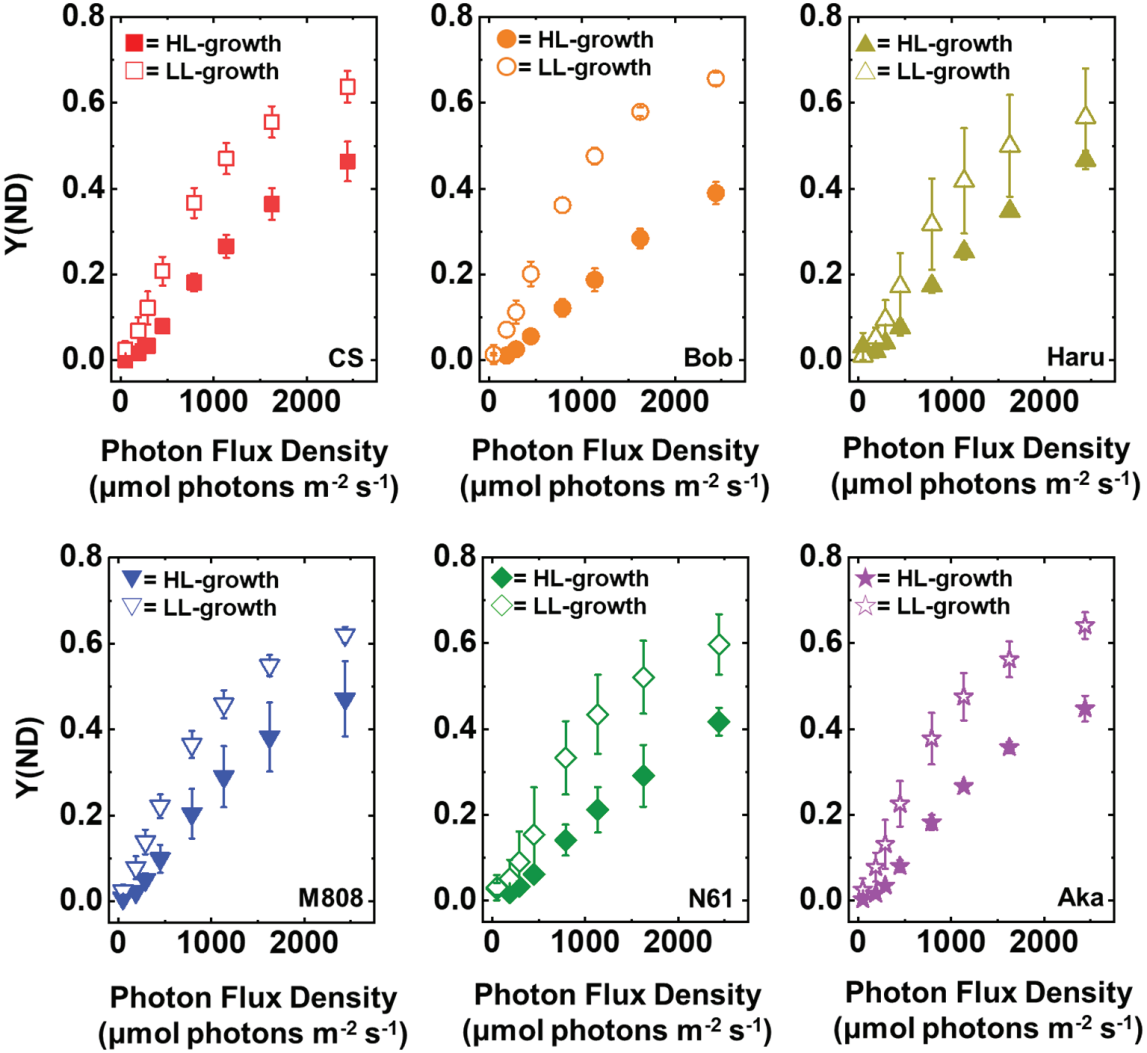
The response of Y(ND) to light intensity in six wheat cultivars grown under LL (open symbol) and HL (closed symbol) conditions. The measurement was conducted under atmospheric conditions (21 kPa O_2_, 40 Pa CO_2_) at 25°C. Data were expressed as mean ± SD of three to five independent experiments. Red squares indicate the results of CS, orange circles indicate the results of Bob, dark yellow triangles indicate the results of Haru, blue inverted-triangles indicate the results of M808, green diamonds indicate the results of N61, and purple stars indicate the results of Aka.

Y(ND) indicates the photosynthetic electron transport limitation at the donor side of PSI, and the increase in Y(ND) indicates the accumulation of oxidized P700 (P700^+^) in PSI (Klughammer and Schreiber, 1994). In LL-grown wheat cultivars, Y(ND) increased from approximately 100 μmol photons m^−2^ s^−1^ of light intensity and showed a saturation of approximately 0.6 at the maximum light intensity (Figure 4). On the other hand, Y(ND) in the HL-grown wheat cultivars hardly increased until the light intensity reached 250 μmol photons m^−2^ s^−1^ (Figure 4). Furthermore, Y(ND) was linearly increased with the rise of illuminated light intensity in the HL-grown wheat cultivars. At the maximum light intensity, the HL-grown wheat plants showed lower Y(ND) than the LL-grown wheat cultivars (Figure 4). A similar effect of growth light conditions on Y(ND) was reported by Schumann et al. (2017). As with the response of Y(I), the significant cultivar differences in Y(ND) in each wheat plant grown under LL and HL conditions were not observed (Supplementary Figures S1E,F).

**Figure 5:**
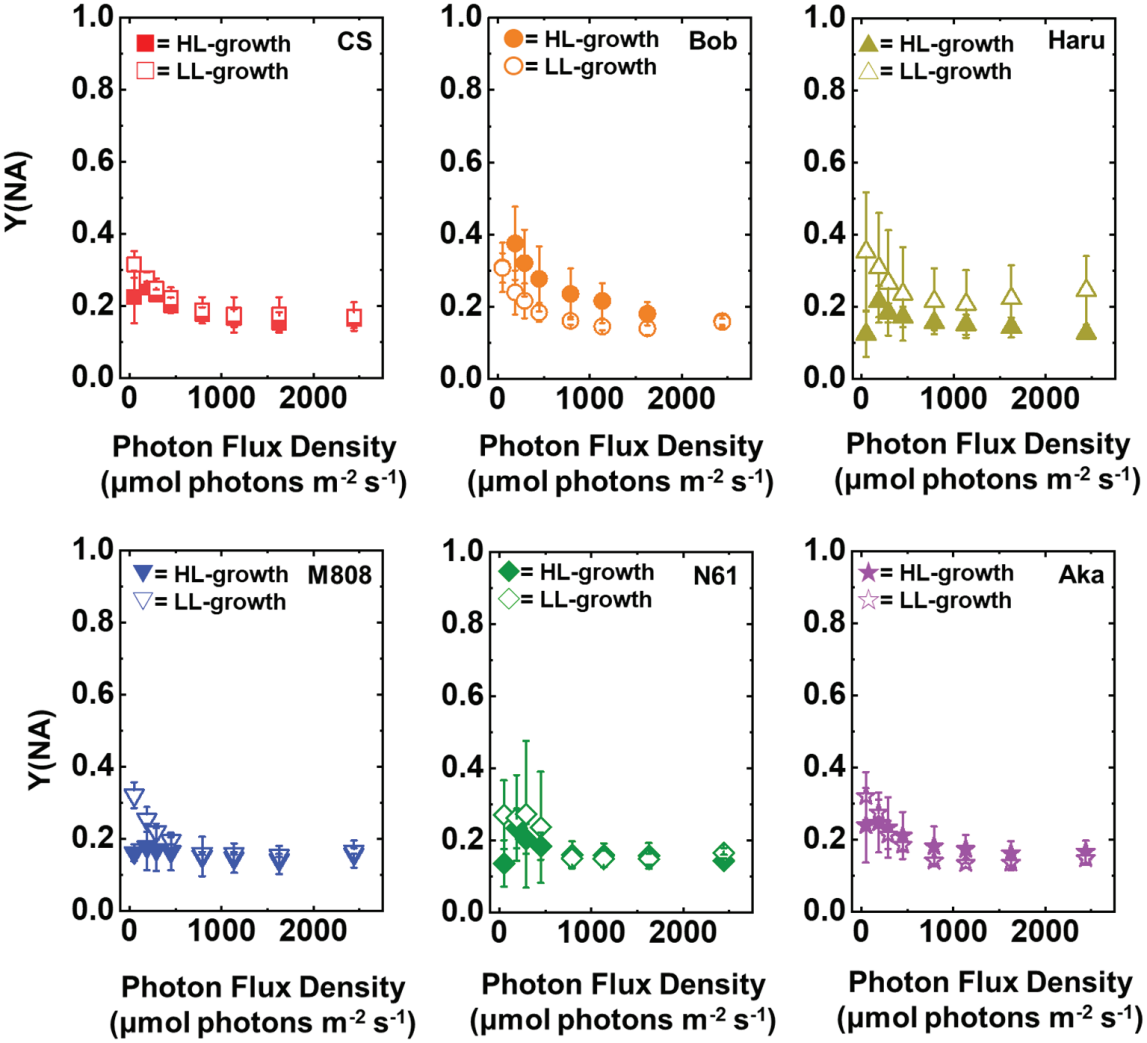
The response of Y(NA) to light intensity in six wheat cultivars grown under LL (open symbol) and HL (closed symbol) conditions. The measurement was conducted under atmospheric conditions (21 kPa O_2_, 40 Pa CO_2_) at 25°C. Data were expressed as mean ± SD of three to five independent experiments. Red squares indicate the results of CS, orange circles indicate the results of Bob, dark yellow triangles indicate the results of Haru, blue inverted-triangles indicate the results of M808, green diamonds indicate the results of N61, and purple stars indicate the results of Aka.

Y(NA) indicates the photosynthetic electron transport limitation at the acceptor side of PSI (Klughammer and Schreiber, 1994). The increase in Y(NA) indicates the risk of ROS production within PSI (Munekage et al., 2002; DalCorso et al., 2008; Suorsa et al., 2012; Shimakawa et al., 2017; Takagi et al., 2017b; Takagi and Miyake, 2018). The light response of Y(NA) was similar among the studied wheat cultivars and growth light conditions even under maximum light intensity, and Y(NA) did not show the cultivar difference in each wheat plant grown under LL and HL conditions (Figure 5, Supplementary Figures S1G,H). These results indicated that the wheat cultivars were capable of maintaining the reduction state of PSI even under different growth light conditions and illuminated light intensities.

### High-Light Acclimation Provides Tolerance to Photosystem I Photoinhibition Depending on Wheat Cultivars and Independently of P700 Oxidation

**Figure 6:**
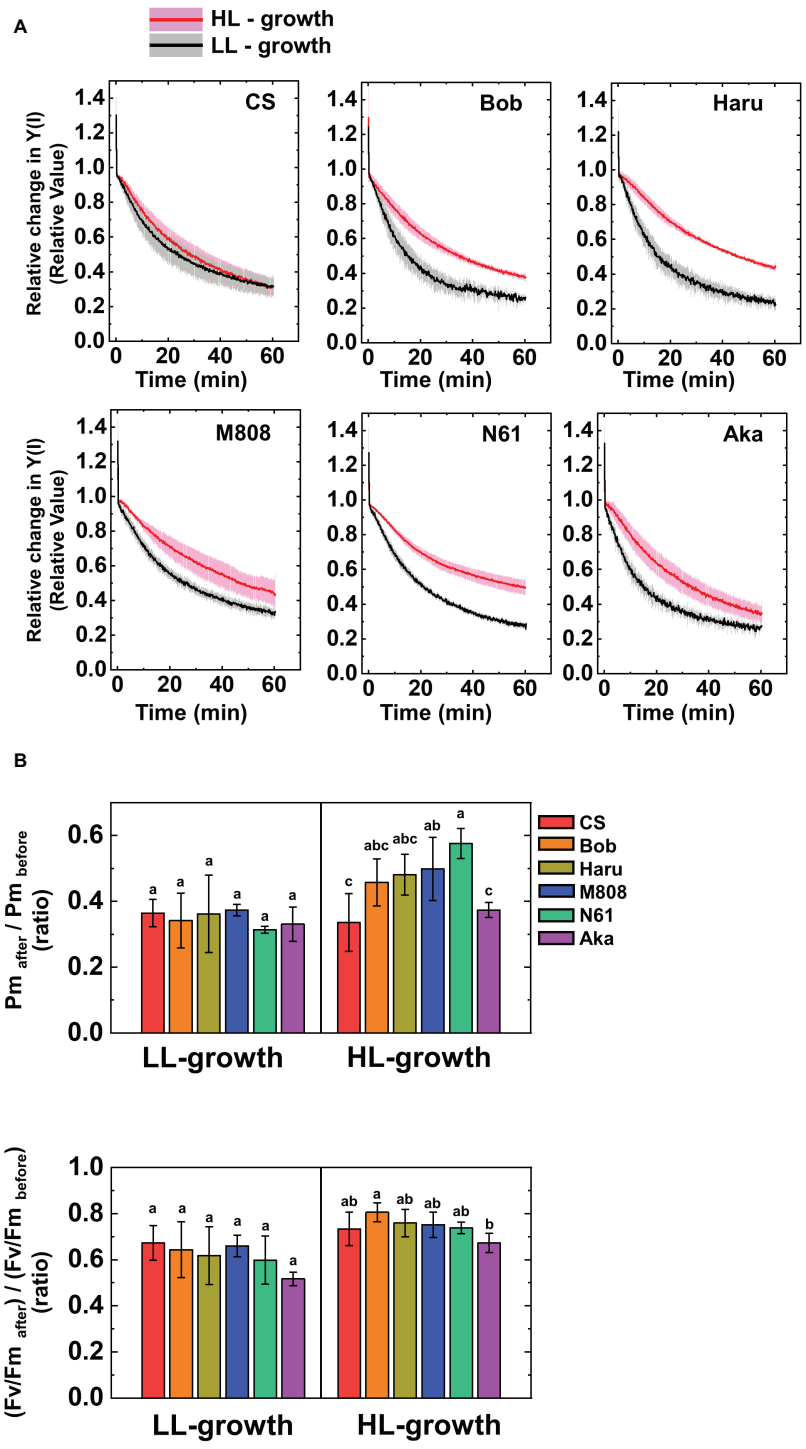
The susceptibility of PSI photoinhibition to repetitive short-pulse (rSP) illumination in wheat cultivars grown under LL and HL conditions. **(A)** Time-course analysis of the relative change in Y(I) during rSP illumination under atmospheric conditions (21 kPa O_2_, 40 Pa CO_2_) at 25°C. Before rSP illumination, wheat plants were adapted in the dark for at least 1 h. Wheat leaves were illuminated every 10 s with a saturating pulse (300 ms, 20,000 μE m^−2^ s^−1^) for 1 h in the dark. Data were expressed as mean ± SD of three to five independent experiments. Black line indicates the mean result of LL-grown wheat cultivars and gray shadow indicates the SD of the results of the LL-grown wheat cultivars. Red line indicates the mean results of the LL-grown wheat cultivars and pink shadow indicates the SD of the results of the HL-grown wheat cultivars. The change in Fv/Fm and Pm between before and after rSP illumination are shown in **(B)**. After rSP illumination, wheat cultivars were kept in the dark for 30 min, and Fv/Fm and Pm were measured. Data were normalized to the Fv/Fm and Pm before rSP illumination. Data were expressed as mean ± SD of three to five independent experiments. Different letters above the bars indicate a significant difference between those wheat cultivars (ANOVA and Tukey-Kramer HSD test, *p* < 0.05). Red bars indicate the results of CS, orange bars indicate the results of Bob, dark yellow bars indicate the results of Haru, blue bars indicate the results of M808, green bars indicate the results of N61, and purple bars indicate the results of Aka.

Next, we studied the effect of growth light conditions on the susceptibility of PSI to its photoinhibition using rSP illumination in the dark under ambient air conditions at 25°C (Sejima et al., 2014; Takagi et al., 2017a). The rSP illumination in the dark stimulates electron accumulation within the thylakoid membranes and causes O_2_-dependent PSI photoinhibition (Sejima et al., 2014; Takagi et al., 2016a, 2017a,b). The relative photoinhibitory effect in PSI during rSP illumination was shown by the relative decrease in Y(I), and the averaged Y(I) for first 1 min was set as “1” according to our previous study (Takagi et al., 2017a). Figure 6A shows the relative change in Y(I) in the dark under ambient air conditions in both LL- and HL-grown wheat cultivars. Interestingly, at the beginning of rSP illumination, we observed the rapid change in relative Y(I) in all wheat cultivars (initial spike; Figure 6A). This phenomenon was also observed in land plants previously (Takagi et al., 2017a). To evaluate whether this initial change in Y(I) indicates the PSI photoinhibition in wheat cultivars, we studied the recovery of this initial change in Y(I) by using CS and M808 grown under LL and HL conditions (Supplementary Figure S2). We confirmed that the rapid change in Y(I) at the beginning of rSP illumination was similar between two wheat cultivars and between LL and HL growth conditions, and the decrease in Y(I) was rapidly recoverable in both LL- and HL-grown wheat cultivars when pulse illumination intervals were prolonged (Supplementary Figure S2). Therefore, the initial change in Y(I) does not show PSI photoinhibition because the recovery of PSI photoinhibition takes several days (Sonoike, 2011; Zivcak et al., 2015). This could indicate the electron accumulation in electron acceptors following P700^+^ by illuminating pulse illumination. It is noteworthy that the decrease in Y(I) during rSP illumination was more suppressed in the HL-grown wheat cultivars compared to the LL-grown ones; however, the effect of HL acclimation varied between the wheat cultivars (Figure 6A). For example, CS showed a similar decrease in Y(I) during rSP illumination in both HL- and LL-grown wheat plants (Figure 6A). On the other hand, the N61 plants grown under HL conditions showed a large alleviation of the decrease in Y(I), compared to those grown under LL conditions (Figure 6A). The relative change in Y(II) during rSP illumination was also studied (Supplementary Figure S3). Similar to the results of Y(I), the decrease in Y(II) was also suppressed in the HL-grown wheat cultivars, compared to that in the LL-grown wheat cultivars (Supplementary Figure S3). The effect of HL acclimation on the change in Y(II) was also cultivar-dependent, but was smaller than that on the change in Y(I). After rSP illumination for 1 h, we evaluated the change in Pm and Fv/Fm in the wheat cultivars grown under HL and LL conditions to study the extent of photoinhibition in both PSI and PSII (Sejima et al., 2014; Takagi et al., 2016a, 2017a). Compared to the Pm before rSP illumination, the steady-state value of oxidized P700 induced by far-red light (FR) was suppressed in all wheat cultivars after rSP illumination for 1 h, and Pm obtained by FR and pulse illumination was also decreased after rSP illumination (Supplementary Figure S4). Among the LL-grown wheat cultivars, the decrease in Fv/Fm and Pm was not different between the wheat cultivars (Figure 6B, Supplementary Figure S4). On the contrary, significant differences were observed among the HL-grown wheat cultivars. N61 showed the smallest decrease in Pm after rSP illumination, and CS showed the largest decrease in Pm after rSP illumination (Figure 6B, Supplementary Figure S4). Furthermore, the HL-grown wheat cultivars, except for CS and Aka, suppressed the decrease in Pm before and after rSP illumination compared to the LL-grown wheat cultivars (Figure 6B). The change in Fv/Fm was also significantly different among the wheat cultivars grown under HL conditions. However, the effect of growth light conditions on the change in Fv/Fm after rSP illumination was smaller than that on the change in Pm after rSP illumination. This is because the rSP illumination mainly targets PSI to induce photoinhibition; therefore, the lesser photoinhibitory effect was observed on PSII in intact leaves (Sejima et al., 2014; Zivcak et al., 2015; Takagi et al., 2016a, 2017b).

**Figure 7:**
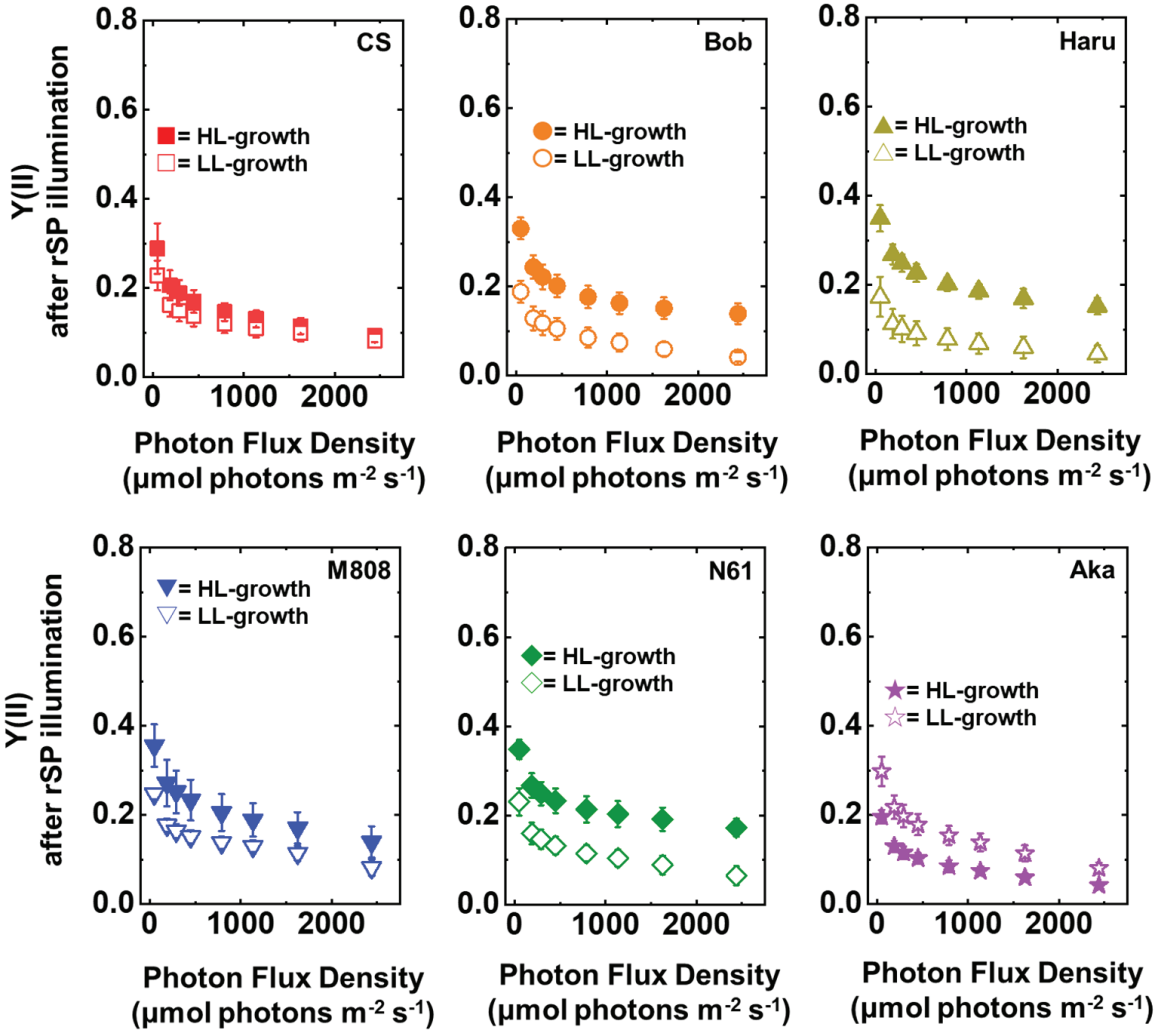
The response of Y(II) to light intensity in six wheat cultivars grown under LL (open symbol) and HL (closed symbol) conditions after rSP illumination for 1 h. The measurement was conducted under atmospheric conditions (21 kPa O_2_, 40 Pa CO_2_) at 25°C. Data are expressed as mean ± SD of three to four independent experiments. Red squares indicate the results of CS, orange circles indicate the results of Bob, dark yellow triangles indicate the results of Haru, blue inverted-triangles indicate the results of M808, green diamonds indicate the results of N61, and purple stars indicate the results of Aka.

After rSP illumination to induce PSI photoinhibition, we measured the photosynthetic activities of the wheat cultivars grown under LL and HL conditions. In both LL- and HL-grown wheat cultivars, the values of Y(II) after rSP illumination were lower than those before rSP illumination (Figure 7). The LL-grown wheat cultivars showed similar values of Y(II) among the studied wheat cultivars; however, significant differences were observed among the HL-grown wheat cultivars, with N61 showing the highest Y(II) and CS showing the lowest Y(II) after rSP illumination (Supplementary Figures S5A,B).

**Figure 8:**
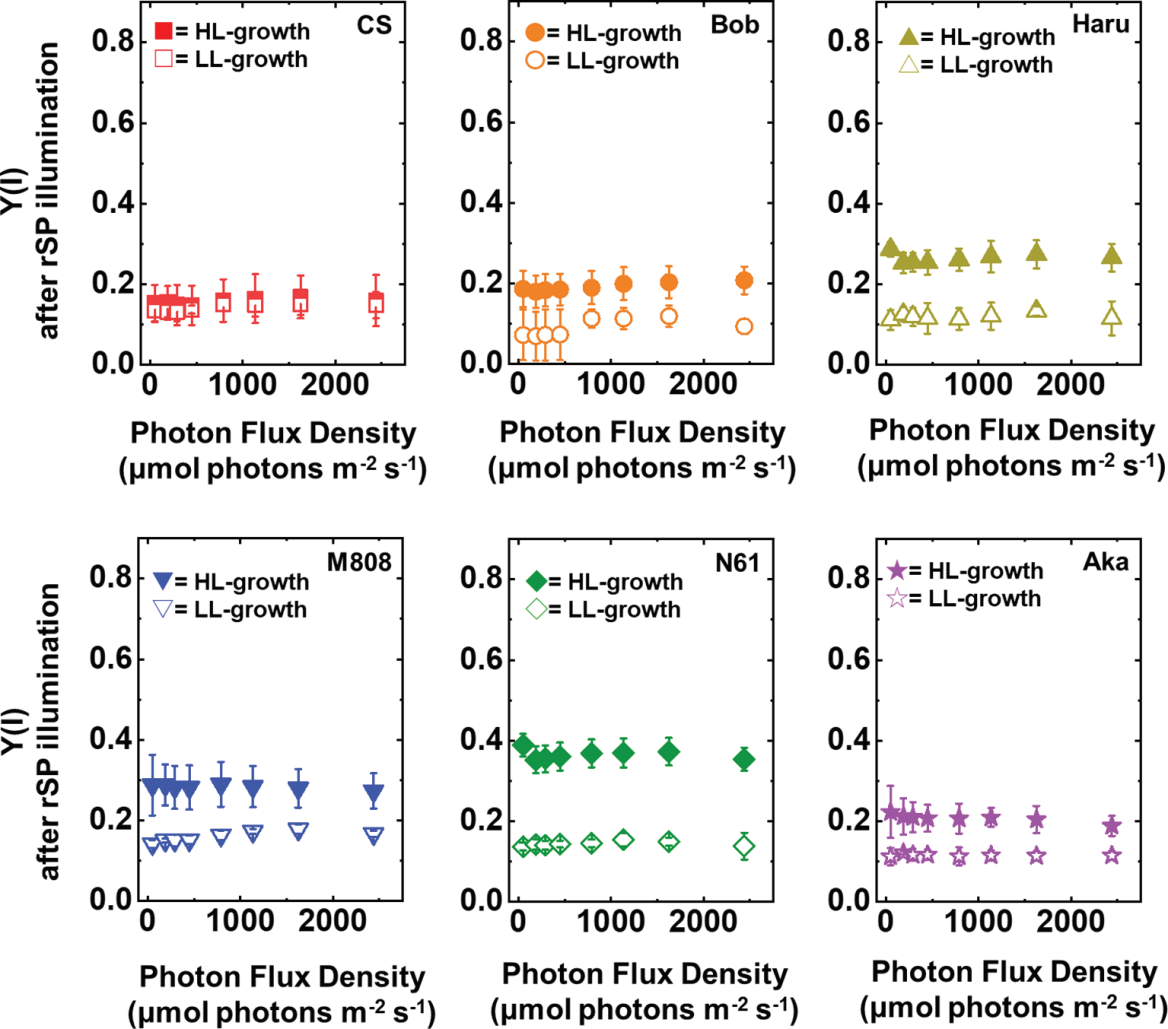
The response of Y(I) to light intensity in six wheat cultivars grown under LL (open symbol) and HL (closed symbol) conditions after rSP illumination for 1 h. The measurement was conducted under atmospheric conditions (21 kPa O_2_, 40 Pa CO_2_) at 25°C. Data are expressed as mean ± SD of three to four independent experiments. Red squares indicate the results of CS, orange circles indicate the results of Bob, dark yellow triangles indicate the results of Haru, blue inverted-triangles indicate the results of M808, green diamonds indicate the results of N61, and purple stars indicate the results of Aka.

Furthermore, the Y(I) value after rSP illumination was significantly lower than that before rSP illumination in all wheat cultivars, hardly showing any change toward the illuminated light intensity (Figure 8). These results agreed with the previous report (Zivcak et al., 2015). Similar to the result of Y(II), the LL-grown wheat cultivars showed similar Y(I) (Figure 8, Supplementary Figures S5C,D). However, among the wheat plants grown under HL conditions, N61 showed the highest Y(I) and CS showed the lowest Y(I) after rSP illumination (Supplementary Figures S5C,D). The results of Y(I) were consistent with those of Y(II) (Figure 7).

**Figure 9:**
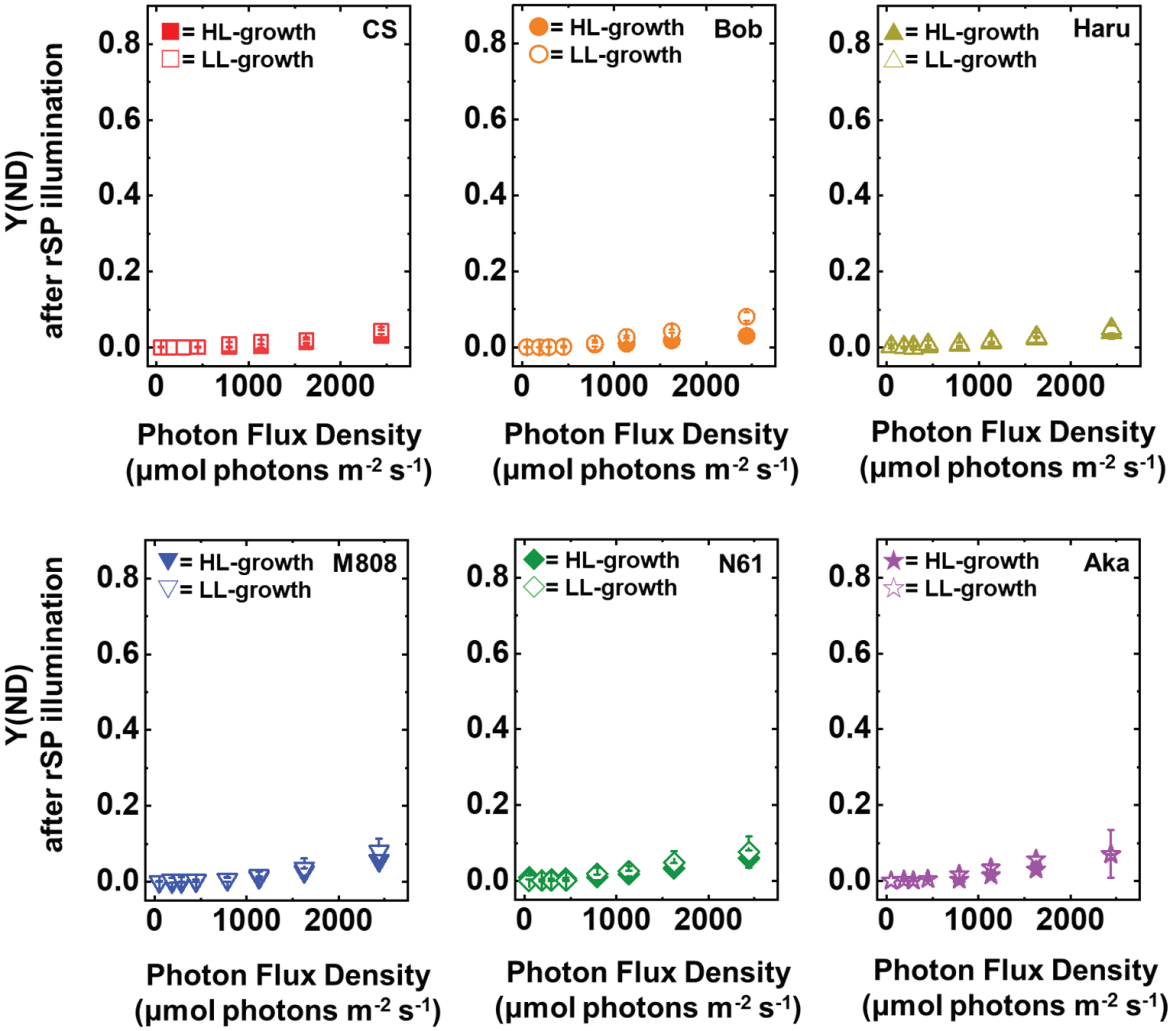
The response of Y(ND) to light intensity in six wheat cultivars grown under LL (open symbol) and HL (closed symbol) conditions after rSP illumination for 1 h. The measurement was conducted under atmospheric conditions (21 kPa O_2_, 40 Pa CO_2_) at 25°C. Data are expressed as mean ± SD of three to four independent experiments. Red squares indicate the results of CS, orange circles indicate the results of Bob, dark yellow triangles indicate the results of Haru, blue inverted-triangles indicate the results of M808, green diamonds indicate the results of N61, and purple stars indicate the results of Aka.

**Figure 10:**
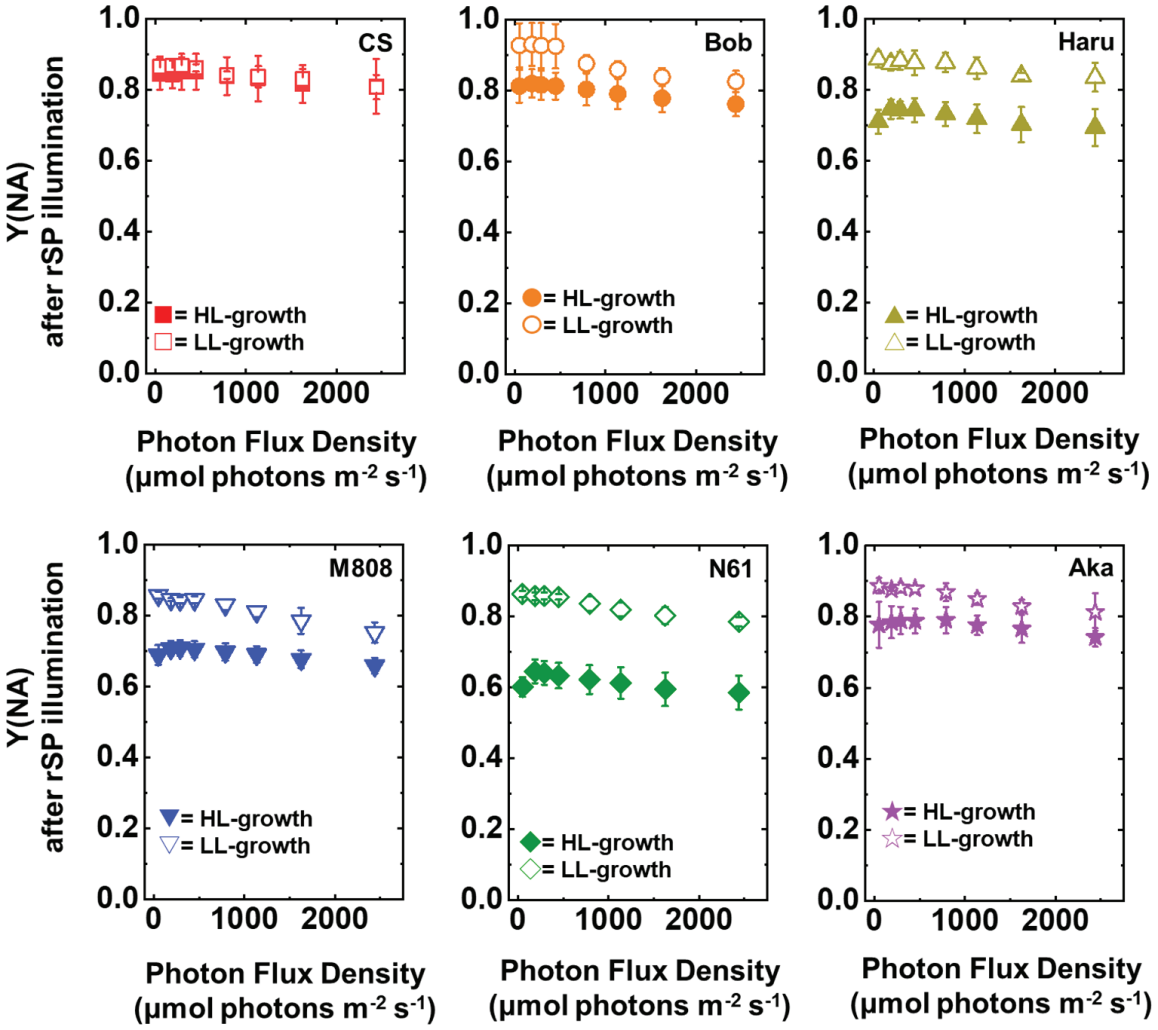
The response of Y(NA) to light intensity in six wheat cultivars grown under LL (open symbol) and HL (closed symbol) conditions after rSP illumination for 1 h. The measurement was conducted under atmospheric conditions (21 kPa O_2_, 40 Pa CO_2_) at 25°C. Data are expressed as mean ± SD of three to four independent experiments. Red squares indicate the results of CS, orange circles indicate the results of Bob, dark yellow triangles indicate the results of Haru, blue inverted-triangles indicate the results of M808, green diamonds indicate the results of N61, and purple stars indicate the results of Aka.

Next, we compared Y(ND) and Y(NA) after rSP illumination in both LL- and HL-grown wheat cultivars (Figures 9, 10). Y(ND) hardly responded to the illuminated light intensity, and the increase in Y(ND) was greatly inhibited (Figure 9). Furthermore, the LL- and HL-grown wheat cultivars showed similar Y(ND) values under illumination (Figure 9, Supplementary Figures S5E,F). Y(NA) in LL-grown wheat cultivars showed similar values after rSP illumination, and the values after rSP illumination were higher than those before rSP illumination (Figure 10, Supplementary Figures S5G,H). These results indicated that rSP illumination triggered the photoinhibition at the acceptor side of PSI and suppressed the photosynthetic electron transport reaction at that site as reported previously (Sejima et al., 2014; Zivcak et al., 2015; Takagi et al., 2016a). Compared to the results of LL-grown wheat cultivars, HL-grown wheat cultivars showed lower Y(NA) and the cultivar differences in Y(NA) were observed among the wheat cultivars after rSP illumination (Figure 10, Supplementary Figures S5G,H). N61 showed the lowest Y(NA) value and CS showed the highest Y(NA) value after rSP illumination (Supplementary Figures S5G,H). These results indicated that N61 showed lower susceptibility of PSI to its photoinhibition compared to CS. To understand the detailed mechanism that rSP illumination caused cultivar difference in Y(I) and Y(NA) among wheat cultivars, we studied the P700^+^ kinetics, from which the Y(I), Y(ND), and Y(NA) were determined (Supplementary Figure S6). As shown in Figure 4, the steady-state level of P700^+^ was higher in LL-grown wheat plants compared to that in HL-grown wheat plants before rSP illumination (Supplementary Figure S6). However, after rSP illumination, the steady-state level of P700^+^ was greatly lowered in both LL- and HL-grown wheat plants, which corresponded to the results of Y(ND) (Figure 9). The cultivar difference in Y(I) and Y(NA) was simply estimated by the difference from the pulse-induced P700^+^ to determine Y(I) and Y(NA) (Supplementary Figure S6).

**Figure 11:**
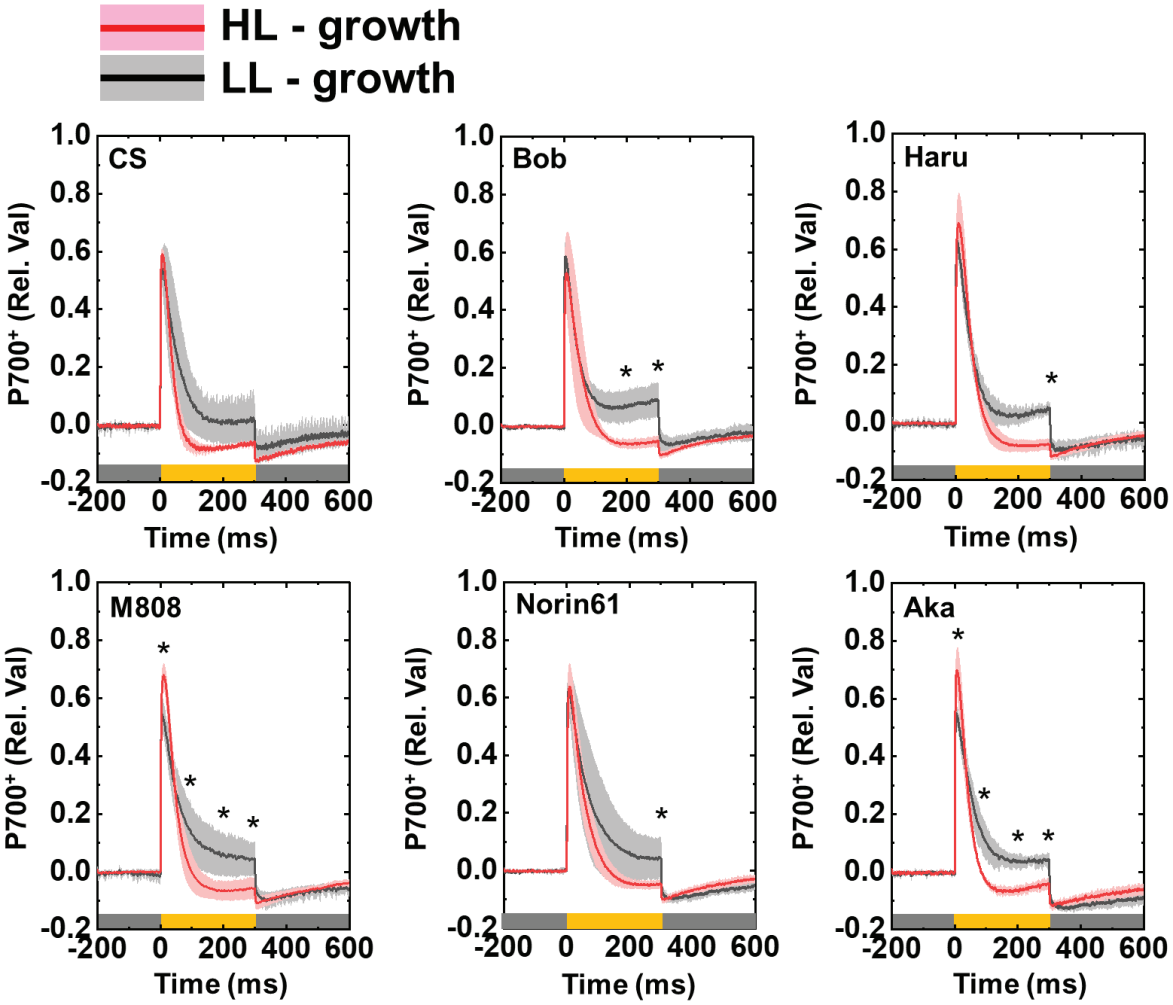
Effect of growth light intensity on the P700^+^ kinetics of wheat cultivars during the one-pulse illumination in rSP illumination. Black lines and gray shadows show the results of wheat cultivars grown under LL conditions, and red lines and pink shadows show the results of wheat cultivars grown under HL conditions. Experiments were performed three times independently, and the data were expressed as mean (solid line) ± SD (shade area). Before measurement, the leaves were kept in the dark for at least 1 h. Pulse illumination (20,000 μmol photons m^−2^ s^−1^, 300 ms) was applied to the leaves every 10 s in the dark at 25°C. Pulse was illuminated from the time “0” in this figure. Dark gray bars indicate the dark conditions and yellow bars indicate the pulse illumination. The difference in the extent of P700^+^ between LL- and HL-grown wheat plants was statistically analyzed at the times 100, 200, and 300 ms during pulse illumination, and peak value (Student’s *t*-test). Asterisks indicate the significant difference in the P700^+^ induction between LL- and HL-grown wheat plants (*p* < 0.05). The cultivar difference in P700^+^ kinetics was also analyzed among LL- and HL-grown wheat cultivars by Tukey-Kramer HSD test; however, no significant difference was detected.

To address whether the susceptibility of PSI to its photoinhibition is determined by P700 oxidation during pulse illumination, we analyzed the P700 oxidation kinetics during one pulse illumination (Takagi et al., 2017a). As with the previous study, we studied the P700 oxidation kinetics at the beginning of rSP illumination to evaluate the effect of P700 oxidation kinetics on PSI photoinhibition (Takagi et al., 2017a). In LL-grown wheat cultivars, the rapid P700 oxidation was observed at the onset of pulse illumination (Figure 11). Subsequently, P700^+^ was reduced even under strong illumination. These results were similar to the results of our previous study on angiosperms (Takagi et al., 2017a). In the HL-grown wheat cultivars, P700 was also rapidly oxidized at the onset of pulse illumination; however, P700^+^ was not maintained in the oxidized state similar to that in the LL-grown wheat cultivars (Figure 11). Importantly, the LL-grown wheat cultivars showed partially higher P700^+^ at the end of pulse illumination (Figure 11). To check the statistical difference in the extent of P700^+^ during pulse illumination, we compared the P700^+^ value at the initial peak and at 100, 200, and 300 ms during the pulse illumination between LL- and HL-grown wheat cultivars. We found that LL-grown wheat plants showed higher P700^+^ especially at the end of pulse illumination (Figure 11). At the same time, we compared the extent of P700^+^ among wheat cultivars grown under LL and HL conditions respectively. Subsequently, we confirmed that P700^+^ kinetics during one pulse illumination did not show cultivar differences among LL- and HL-grown wheat cultivars respectively (ANOVA, *p* > 0.05). Therefore, the HL acclimation response and cultivar differences in susceptibility to PSI photoinhibition cannot be attributed to the P700 oxidation during pulse illumination.

## Discussion

When land plants grow under LL intensity, they develop light-harvesting mechanisms for effective light absorption (De la Torre and Burkey, 1990; Bailey et al., 2001; Oguchi et al., 2003; Walters et al., 2003; Dietz, 2015; Bielczynski et al., 2016; Schumann et al., 2017). This is because photosynthesis is limited by light absorption under LL conditions (Roach and Krieger-Liszkay, 2014). On the other hand, for surviving under the strong light environment, land plants improved their electron sink capacities and photoprotective mechanisms in PSII (Miyata et al., 2015; Ware et al., 2015; Schumann et al., 2017). These responses contribute to escaping excessive electron accumulation in the photosynthetic electron transport chain and reducing the risk of ROS production and oxidative damage in chloroplasts. In this study, we characterized the effect of HL acclimation on the susceptibility of PSI to its photoinhibition by using rSP illumination (Sejima et al., 2014; Takagi et al., 2016a). Subsequently, we found that HL acclimation lowered the susceptibility of PSI to PSI photoinhibition depending on wheat cultivars (Figure 6). In general, the photosynthetic electron transport activity exceeds the electron sink capacity under HL illumination (Roach and Krieger-Liszkay, 2014). The excessive accumulation of electrons on the thylakoid membranes stimulates ROS production within PSI and causes oxidative damage to the photosynthetic apparatus in order to inhibit CO_2_ fixation (Zivcak et al., 2015). A decrease in the susceptibility of PSI to its photoinhibition would be a physiological mechanism to escape the risk of ROS-dependent oxidative damage. Until now, the effects of growth light conditions on PSI photoprotective mechanisms have been rarely studied (Sonoike et al., 1995a,b; Sonoike, 1996a, 1998; Ivanov et al., 1998), although the effects of HL acclimation on the photoprotective mechanisms in PSII have been greatly focused on. This would be due to the difficulty in evaluating the susceptibility to PSI photoinhibition compared to that of PSII. As discussed later, the effects of rSP illumination on PSI photochemistry and the chilling treatment on PSI photochemistry certainly resembled. Based on these facts, we suggest the possibility that rSP illumination would reproduce the effect of chilling stress on PSI photoinhibition independent of chilling temperature. Therefore, the results of this study would provide the opportunity to analyze the relationship between the photoprotective mechanisms of PSI and growth light conditions in the future.

The susceptibility of PSI to its photoinhibition was not differentiated between the spring-type and winter-type wheat plants. Several studies reported that the winter-type wheat plants showed an increase in photoprotective mechanisms in PSII by acclimating to HL or cold temperature (Hurry et al., 1995; Gray et al., 1996, 1997; Savitch et al., 2000; Pocock et al., 2001). However, we found no clear difference in the HL acclimation response to the susceptibility of PSI to its photoinhibition between the spring-type and winter-type wheat cultivars (Figure 6). In general, a difference of the two growth habits, spring-type and winter-type, is determined by vernalization requirement for a transition from the vegetative phase to the reproductive phase in wheat, and this phenotypic difference is mainly controlled by *Vrn1* on the long arms of group 5 chromosomes (Fu et al., 2005). Genetic factors controlling frost and freezing tolerance might be present in the *Vrn-1* chromosomal regions (Galiba et al., 2009), and the winter-type wheat cultivars should provide frost and freezing tolerance. It is still under discussion whether *Vrn-1* itself could be a regulator of frost and freezing tolerance in wheat. The results of this study indicated that the *Vrn-1* allelic differences between the spring-type and winter-type wheat cultivars are irrelevant to the acclimation response of PSI photoprotective mechanisms.

The photoinhibitory effects of rSP illumination would reproduce the PSI photoinhibition in chilling-sensitive plants under chilling treatment. Havaux and Davaud (1994) reported that chilling-stressed potato leaves decrease the P700 oxidation efficiency by illuminating FR light in addition to the maximum oxidizable P700, and the Emerson enhancement of photosynthetic O_2_ evolution is stimulated compared to non-chilling stressed leaves. Sonoike (1999) also showed a similar result by using cucumber leaves *in vivo,* and P700 oxidation was much slower under FR light illumination compared to non-stressed cucumber leaves. These results indicated that the quantum efficiency in PSI is lowered after PSI photoinhibition in chilling-sensitive plants by chilling treatment besides the decrease in P700 content. Here, we also observed the decrease in the light responsiveness of Y(I) after rSP illumination in all wheat leaves (Figure 8; Zivcak et al., 2015). These results correspond to the lower quantum efficiency in PSI (Terao and Katoh, 1996). Moreover, the quantum efficiency evaluated from dichlorophenolindophenol (DCIP) and methyl-viologen (MV)-dependent O_2_ absorption by PSI is also lowered in isolated chloroplasts causing PSI photoinhibition by rSP illumination (Takagi et al., 2016a). Based on these observations, the effects of chilling stress on PSI in chilling-sensitive plants and the effect of rSP illumination on PSI in land plants could be similar. In the previous reports, the decrease in the quantum efficiency in PSI has been explained as the stimulation of charge recombination reaction due to the higher acceptor side electron transport limitation (Havaux and Davaud, 1994) and the stimulation of CEF-PSI (Sonoike, 1999). In addition to those, the possibility that the antenna system of PSI is injured during PSI photoinhibition cannot be discarded (Scheller and Haldrup, 2005; Benson et al., 2015; Yamatani et al., 2018). Kok et al. (1965) showed that 77 K fluorescence emission corresponding to PSI-light harvesting complex (LHC) I was decreased after PSI photoinhibition. Furthermore, Rajagopal et al. (2005) reported that LHCI is target by ROS produced in PSI, and Tiwari et al. (2016) reported that the direct light absorption by PSI-core but not by LHCI is increased during the progress of PSI photoinhibition by using PROTON GRADIENT REGULATION 5 mutant (*pgr5*) in *Arabidopsis thaliana* which is susceptible to PSI photoinhibition (Munekage et al., 2002). Here we should note that we only evaluated the PSI photoinhibition by absorbance change in P700^+^ based on non-stressed leaves, and we calculated the redox parameters of PSI after rSP illumination based on the Pm before rSP illumination (see Materials and Methods). In this case, we cannot distinguish the cause of the change in PSI redox parameters by a qualitative change in electron transport within PSI (Sonoike et al., 1995a,b; Tiwari et al., 2016) or a quantitative change in PSI-LHCI complex and its subunits (Sonoike, 1996b; Tjus et al., 1999). In this study, we cannot conclude the cause of lowering the quantum yield of PSI after PSI photoinhibition. However, the general aspect of PSI photoinhibition to PSI photochemistry now can be addressed by using rSP illumination.

We proposed that two independent mechanisms operate for protecting PSI from its ROS-triggered photoinhibition. Recently, we suggested that the P700 oxidation system is important for reducing the risk of PSI photoinhibition in various photosynthetic organisms (Shimakawa et al., 2016, 2017, 2019; Takagi et al., 2016b, 2017a; Takagi and Miyake, 2018). The stimulation of P700 oxidation by P700 oxidation systems is achieved by limiting electron transport from PSII to PSI or stimulating an outflow of electrons from PSI (Shimakawa et al., 2016, 2017, 2019; Takagi et al., 2016b, 2017a). In this study, we analyzed the P700 oxidation kinetics during pulse illumination (Figure 11). However, compared to HL-grown wheat cultivars, LL-grown wheat cultivars showed higher P700^+^ during one pulse illumination, although the change in Pm after rSP illumination was greater in LL-grown wheat plants than HL-grown wheat plants (Figure 6). Moreover, we cannot detect the significant difference in the extent of P700^+^ during one pulse illumination between wheat cultivars grown under HL conditions (Figure 11). Therefore, the susceptibility of PSI to its photoinhibition cannot be explained by P700^+^ oxidation. The effect of HL acclimation on the susceptibility of PSI photoinhibition evaluated by rSP illumination agrees with the previous reports which showed the acclimation response of the susceptibility of PSI to its photoinhibition by using chilling stress and chilling-sensitive plants (Sonoike et al., 1995a,b; Sonoike, 1996a; Ivanov et al., 1998). Ivanov et al. (1998) proposed that the susceptibility of PSI to its photoinhibition depends on the down-regulation of PSII activity. This is a novel PSI protection mechanism to avoid the electron accumulation within PSI (Sonoike, 1996b; Tikkanen et al., 2014). However, during rSP illumination, wheat plants which maintained higher Pm after rSP illumination also maintained higher Y(II) during rSP illumination, and the change in Fv/Fm was also minor (Figure 6B, Supplementary Figure S3). Therefore, although the acclimation response of the susceptibility of PSI to its photoinhibition identified in this study is similar to previous reports (Ivanov et al., 1998), the protection mechanism of PSI which can be addressed by rSP illumination would be different from those addressed by chilling treatment. On the basis of P700^+^ kinetics during pulse illumination, the electron accumulation within PSI would be higher in HL-grown wheat cultivars than that in LL-grown wheat cultivars during rSP illumination (Figure 11). When we focused on the cause of PSI photoinhibition attributing to ${O_{2}}^{-}$, the difference in the susceptibility of PSI to photoinhibition should be caused by ${O_{2}}^{-}$ production efficiency or the ${O_{2}}^{-}$ scavenging activity within the thylakoid membranes. On the other hand, the production of ^1^O_2_ within PSI cannot be monitored from the P700 oxidation kinetics. In fact, a ^1^O_2_ scavenger suppresses PSI photoinhibition induced by rSP illumination without affecting the P700^+^ kinetics during pulse illumination in isolated chloroplasts (Takagi et al., 2016a). The suppression of the charge separation from P700 to P700^+^ during pulse illumination increases the risk that excited P700 (*P700) within PSI triggers ^1^O_2_ production through ^3^P700 (Takagi et al., 2016a). From this view, we additionally suggest that the difference in the susceptibility of PSI to its photoinhibition might be determined by the ^1^O_2_-scavenging or quenching mechanisms of *P700 or ^3^P700 to suppress the production of ^1^O_2_ (Telfer et al., 1994; Rajagopal et al., 2005; Cazzaniga et al., 2012; Dall’Osto et al., 2012; Ballottari et al., 2014). In summary, we propose a new photoprotective mechanism in PSI whose capacity is modulated by growth light environment, and this PSI photoprotective mechanism would cooperate with P700 oxidation system to reduce the risk of PSI photoinhibition caused by ROS.

**Figure 12:**
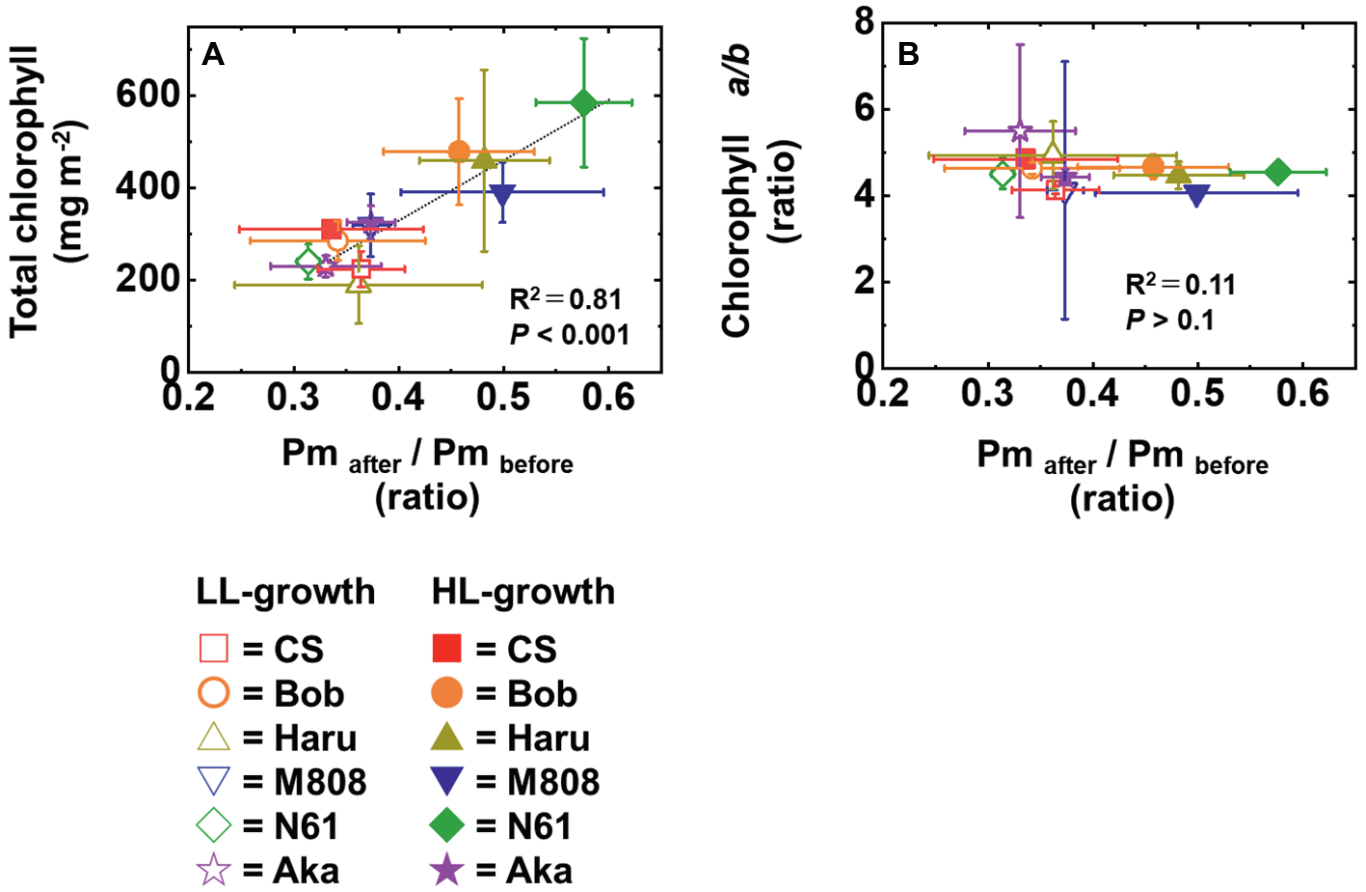
The relationship between the change in Pm by rSP illumination for 1 h and total chlorophyll content in leaves **(A)** and between the change in Pm by rSP illumination for 1 h and chlorophyll *a*/*b* ratio **(B)**. The data of the change in Pm after rSP illumination were used from Figure 6B, the data of the total chlorophyll content were used from Figure 1B, and the data of the total chlorophyll *a*/*b* ratio were used from Figure 1C. Data were expressed as mean ± SD of three to five independent experiments. *R*^2^ represents the determination coefficient of the linear regression analysis. *R*^2^ and *p* were determined by fitting a linear regression model (see section Materials and methods). The linear regression line determined by the statistical analysis is shown as the black broken line.

We suggest that leaf chlorophyll content would be one important determinant of the susceptibility of PSI to its photoinhibition. Unfortunately, we cannot distinguish the susceptibility of PSI to its photoinhibition from the acclimation response of Y(II), Y(I), Y(ND), and Y(NA). This is because all wheat cultivars showed a similar change in these photosynthetic parameters toward the LL and HL growth conditions (Figures 2–5, Supplementary Figure S1). We also examined the non-photochemical quenching (NPQ) in both LL- and HL-grown wheat cultivars (Supplementary Figure S7). In LL-grown wheat plants, all wheat cultivars showed similar NPQ in the light curve analysis (Supplementary Figure S7). HL-grown wheat cultivars showed lower NPQ in the light curve analysis compared to LL-grown wheat cultivars, and also showed cultivar differences in the light curve analysis (Supplementary Figure S7). Generally, electron sink capacity is increased when land plants grow under HL conditions as observed in Figures 2, 3 (Bailey et al., 2001; Oguchi et al., 2003; Dahal et al., 2012; Schumann et al., 2017). Therefore, the induction of NPQ would be lowered in HL-grown wheat cultivars compared to LL-grown wheat cultivars under strong illumination (Ballottari et al., 2007). In addition to the change in electron sink capacity during HL acclimation, PSII:PSI ratio, PSII:LHCII ratio, and the protein contents which are involved in NPQ like PsbS, violaxanthin de-epoxidase (VDE), and zeaxanthin epoxidase (ZE) are also changed depending on the growth light (Bailey et al., 2001; Ballottari et al., 2007; Kouřil et al., 2013). These changes might be caused by the cultivar differences grown under HL conditions. In spite of the cultivar difference in NPQ in the light curve analysis, NPQ induction was not different between wheat cultivars during rSP illumination (Supplementary Figure S8). During rSP illumination, NPQ was slowly induced (Supplementary Figure S8). This would be due to the progress of PSII photoinhibition as observed in the decrease in Y(II) during rSP illumination (Supplementary Figure S3). From these observations, we suggest that the susceptibility of PSI to its photoinhibition cannot be determined by the NPQ kinetics in the light curve analysis and during rSP illumination. With the responses of photosynthetic parameters to the growth light conditions, the total chlorophyll contents and chlorophyll *a*/*b* ratio showed significant differences among the wheat cultivars grown under HL conditions (Figure 1). Based on this observation, we plotted the relationship between the total chlorophyll contents, chlorophyll *a*/*b* ratio, and the susceptibility of PSI to photoinhibition. Subsequently, we observed a positive linear relationship between the total chlorophyll content and the residual activity of PSI after rSP illumination, although the chlorophyll *a*/*b* ratio showed no significant relationship to the residual activity of PSI after rSP illumination (Figure 12). Therefore, the light acclimation response of the susceptibility of PSI to photoinhibition would be changed with the change in chlorophyll content in leaves in wheat cultivars. Such correlation has not been observed before (Sonoike et al., 1995a,b). This would be a hint for elucidating the molecular mechanism to determine the susceptibility of PSI to photoinhibition for future research. Alboresi et al. (2009) reported that the susceptibility of PSI to its photoinhibition depends on the PSI-LHCI complex formation. The binding of carotenoids in LHCI is also an important factor to determine the ROS production (Cazzaniga et al., 2012; Dall’Osto et al., 2012; Ballottari et al., 2014). As discussed above, LHCI is a possible target of PSI photoinhibition. Therefore, LHCI is likely involved in PSI photoinhibition. Furthermore, because the chlorophyll *a*/*b* ratio is higher in LHCI (1.8–5.5) than in LHCII (1.15–1.5) (Hemelrijk et al., 1992; Morosinotto et al., 2003), the quantity of LHCI hardly affects the leaf chlorophyll *a*/*b* ratio (Wientjes et al., 2009; Yamatani et al., 2018). If our hypothesis mentioned above is correct, exploration of the interaction between susceptibility to PSI photoinhibition, LHCI content and energy transfer from LHCI to PSI core should certainly lead to the elucidation of the detailed mechanisms of the PSI photoinhibition *in planta.*

In addition to the change in the susceptibility of PSI to its photoinhibition in leaves, we found that the wheat plants grown under both LL and HL conditions keep Y(NA) low and maintain similar values under the illumination (Figure 5). The value of Y(NA) is determined by the balance of electron inflow and outflow within PSI (Klughammer and Schreiber, 1994; Shimakawa et al., 2016, 2017, 2019; Takagi et al., 2016b, 2017a; Takagi and Miyake, 2018). In other words, when wheat plants possess electron sink capacities lower than photosynthetic electron transport reactions, they build up Y(ND) to decrease Y(NA) (Figures 4, 5; Takagi et al., 2016b, 2017b; Wada et al., 2018). On the other hand, when wheat plants possess large electron sink capacities, they relax Y(ND) and stimulate energy production for driving photosynthesis as can be judged from the change in Y(I) (Figure 3; Takagi et al., 2016b, 2017b; Wada et al., 2018). This would be modulated by P700 oxidation systems (Shimakawa et al., 2016, 2017, 2019; Takagi et al., 2016b, 2017a; Takagi and Miyake, 2018). We propose that a P700 oxidation system could respond to growth light intensity and flexibly control the redox state of PSI at the steady state photosynthesis to reduce the risk of ROS.

Here, we proposed that rSP illumination is a useful method to elucidate the mechanism of PSI photoinhibition *in vivo*. In the present study, rSP illumination showed an HL acclimation effect on PSI photoinhibition and cultivar differences in PSI photoinhibition after HL acclimation among the studied wheat cultivars. To our knowledge, this is the first study that showed the growth light acclimation response of the susceptibility of PSI to its photoinhibition by using rSP illumination. Therefore, rSP illumination enables the researchers to explore the new world of PSI photoprotection mechanisms by applying rSP illumination to various plant species more easily. At this stage, we have not yet identified the key factor underlying the difference in PSI photoinhibition among the studied wheat cultivars. However, it would be possible to isolate the causative gene by the forward genetic experiments like the quantitative trait locus (QTL) analysis combined with rSP illumination because the HL acclimation response of the susceptibility of PSI showed cultivar differences. We also suggest the combined analysis of rSP illumination and chilling treatment would contribute to elucidating the general protection mechanisms of PSI to its photoinhibition. Under chilling treatment, PSI photoinhibition can be easily caused by low light illumination in intact leaves (Sonoike and Terashima, 1994; Terashima et al., 1994; Sonoike, 1999). At that time, various changes in the temperature-dependent biochemical reaction would cause the susceptibility of PSI to its photoinhibition (ex. Lipid fluidity or enzyme stabilities/activities) (Sonoike, 1996a, 1998). On the other hand, rSP illumination can cause PSI photoinhibition under room temperature. The two PSI photoinhibitory treatments could distinguish photoprotective mechanism of PSI, which is involved in temperature-dependent components or temperature-independent components. For example, under chilling treatment, the uncoupling of chloroplastic ATP synthase stimulates the PSI photoinhibition through the over-reduction of PSI by suppressing ΔpH-dependent photosynthetic electron transport regulation (Terashima et al., 1991a,b). In contrast, PSI photoinhibition induced by rSP illumination in the dark does not affect the ΔpH formation ability because short pulse cannot build up enough ΔpH during rSP illumination under the dark conditions (Takagi et al., 2017b). We hope that our present results trigger a wide range of interest in PSI photoinhibition as well as PSII photoinhibition and the key factor to be identified in the future.

## Author Contributions

DT, ST, and CM contributed to the conceptualization of this study. DT and HI carried out investigations. DT and ST prepared the original draft. DT, HI, ST, and CM wrote, reviewed, and edited the manuscript. CM contributed to funding acquisition.

### Conflict of Interest Statement

The authors declare that the research was conducted in the absence of any commercial or financial relationships that could be construed as a potential conflict of interest.

## References

[ref1] Alboresi A. Ballottari M. Hienerwadel R. Giacometti G. M. Morosinotto T. (2009). Antenna complexes protect photosystem I from photoinhibition. BMC Plant Biol. 9:71. 10.1186/1471-2229-9-71, PMID: 19508723 PMC2704212

[ref2] Alter P. Dreissen A. Luo F. L. Matsubara S. (2012). Acclimatory responses of *Arabidopsis* to fluctuating light environment: comparison of different sunfleck regimes and accessions. Photosynth. Res. 113, 221–237. 10.1007/s11120-012-9757-2, PMID: 22729524 PMC3430843

[ref3] Atkin O. K. Tjoelker M. G. (2003). Thermal acclimation and the dynamic response of plant respiration to temperature. *Trends Plant Sci*. 8, 343–351. 10.1016/S1360-1385(03)00136-5, PMID: 12878019

[ref4] Bailey S. Walters R. G. Jansson S. Horton P. (2001). Acclimation of *Arabidopsis thaliana* to the light environment: the existence of separate low light and high light responses. Planta 213, 794–801. 10.1007/s004250100556, PMID: 11678285

[ref5] Baker N. R. (2008). Chlorophyll fluorescence: a probe of photosynthesis *in vivo*. Annu. Rev. Plant Biol. 59, 89–113. 10.1146/annurev.arplant.59.032607.092759, PMID: 18444897

[ref6] Ballottari M. Alcocer M. J. D’Andrea C. Viola D. Ahn T. K. Petrozza A. (2014). Regulation of photosystem I light harvesting by zeaxanthin. Proc. Natl. Acad. Sci. USA 111, E2431–E2438. 10.1073/pnas.1404377111 24872450 PMC4060657

[ref7] Ballottari M. Dall’Osto L. Morosinotto T. Bassi R. (2007). Contrasting behavior of higher plant photosystem I and II antenna systems during acclimation. *J. Biol. Chem*. 282, 8947–8958. 10.1074/jbc.M606417200, PMID: 17229724

[ref8] Benson S. L. Maheswaran P. Ware M. A. Hunter C. N. Horton P. Jansson S.. (2015). An intact light harvesting complex I antenna system is required for complete state transitions in *Arabidopsis*. Nat. Plants 1:15176. 10.1038/nplants.2015.176, PMID: 27251716

[ref9] Bielczynski L. W. Schansker G. Croce R. (2016). Effect of light acclimation on the organization of photosystem II super- and sub-complexes in *Arabidopsis thaliana*. Front. Plant Sci. 7:105. 10.3389/fpls.2016.00105, PMID: 26925068 PMC4756287

[ref10] Cazzaniga S. Li Z. Niyogi K. K. Bassi R. Dall’Osto L. (2012). The *Arabidopsis szl1* mutant reveals a critical role of β-carotene in photosystem I photoprotection. Plant Physiol. 159, 1745–1758. 10.1104/pp.112.201137, PMID: 23029671 PMC3425210

[ref11] Cohu C. M. Muller O. Adams W. W. Demmig-Adams B. (2014). Leaf anatomical and photosynthetic acclimation to cool temperature and high light in two winter versus two summer annuals. Physiol. Plant. 152, 164–173. 10.1111/ppl.12154, PMID: 24450735

[ref12] Dahal K. Kane K. Gadapati W. Webb E. Savitch L. V. Singh J.. (2012). The effects of phenotypic plasticity on photosynthetic performance in winter rye, winter wheat and *Brassica napus*. Physiol. Plant. 144, 169–188. 10.1111/j.1399-3054.2011.01513.x, PMID: 21883254

[ref13] DalCorso G. Pesaresi P. Masiero S. Aseeva E. Schünemann D. Finazzi G.. (2008). A complex containing PGRL1 and PGR5 is involved in the switch between linear and cyclic electron flow in *Arabidopsis*. Cell 132, 273–285. 10.1016/j.cell.2007.12.028, PMID: 18243102

[ref14] Dall’Osto L. Holt N. E. Kaligotla S. Fuciman M. Cazzaniga S. Carbonera D.. (2012). Zeaxanthin protects plant photosynthesis by modulating chlorophyll triplet yield in specific light-harvesting antenna subunits. J. Biol. Chem. 287, 41820–41834. 10.1074/jbc.M112.405498, PMID: 23066020 PMC3516730

[ref15] De la Torre W. R. Burkey K. O. (1990). Acclimation of barley to changes in light intensity: photosynthetic electron transport activity and components. Photosynth. Res. 24, 127–136. 10.1007/BF00032593, PMID: 24419906

[ref16] Dietz K. J. (2015). Efficient high light acclimation involves rapid processes at multiple mechanistic levels. J. Exp. Bot. 66, 2401–2414. 10.1093/jxb/eru505, PMID: 25573858

[ref17] Dumlao M. R. Darehshouri A. Cohu C. M. Muller O. Mathias J. Adams W. W. III. (2012). Low temperature acclimation of photosynthetic capacity and leaf morphology in the context of phloem loading type. Photosynth. Res. 113, 181–189. 10.1007/s11120-012-9762-5, PMID: 22791016

[ref18] Ensminger I. Busch F. Huner N. (2006). Photostasis and cold acclimation: sensing low temperature through photosynthesis. Physiol. Plant. 126, 28–44. 10.1111/j.1399-3054.2006.00627.x, PMID: 23230444

[ref19] Fu D. Szűcs P. Yan L. Helguera M. Skinner J. S. Von Zitzewitz J.. (2005). Large deletions within the first intron in *VRN-1* are associated with spring growth habit in barley and wheat. Mol. Gen. Genomics. 273, 54–65. 10.1007/s00438-004-1095-4, PMID: 15690172

[ref20] Galiba G. Vágújfalvi A. Li C. Soltész A. Dubcovsky J. (2009). Regulatory genes involved in the determination of frost tolerance in temperate cereals. Plant Sci. 176, 12–19. 10.1016/j.plantsci.2008.09.016

[ref21] Goldschmidt-Clermont M. Roberto B. (2015). Sharing light between two photosystems: mechanism of state transitions. Curr. Opin. Plant Biol. 25, 71–78. 10.1016/j.pbi.2015.04.009, PMID: 26002067

[ref22] Gray G. R. Chauvin L. P. Sarhan F. Huner N. P. (1997). Cold acclimation and freezing tolerance (a complex interaction of light and temperature). Plant Physiol. 114, 467–474. 10.1104/pp.114.2.467, PMID: 12223720 PMC158326

[ref23] Gray G. R. Savitch L. V. Ivanov A. G. Huner N. P. (1996). Photosystem II excitation pressure and development of resistance to photoinhibition (II. Adjustment of photosynthetic capacity in winter wheat and winter rye). Plant Physiol. 110, 61–71. 10.1104/pp.110.1.61, PMID: 12226171 PMC157694

[ref24] Havaux M. Davaud A. (1994). Photoinhibition of photosynthesis in chilled potato leaves is not correlated with a loss of photosystem-II activity. Photosynth. Res. 40, 75–92. 10.1007/BF00019047, PMID: 24311216

[ref25] Hemelrijk P. W. Kwa S. L. van Grondelle R. Dekker J. P. (1992). Spectroscopic properties of LHC-II, the main light-harvesting chlorophyll *a*/*b* protein complex from chloroplast membranes. Biochim. Biophys. Acta Bioenerg. 1098, 159–166. 10.1016/S0005-2728(05)80331-7

[ref26] Hüner N. P. Dahal K. Kurepin L. V. Savitch L. Singh J. Ivanov A. G.. (2014). Potential for increased photosynthetic performance and crop productivity in response to climate change: role of CBFs and gibberellic acid. *Front. Chem*. 2:18. 10.3389/fchem.2014.00018, PMID: 24860799 PMC4029004

[ref27] Hüner N. Öquist G. Sarhan F. (1998). Energy balance and acclimation to light and cold. Trends Plant Sci. 3, 224–230. 10.1016/S1360-1385(98)01248-5

[ref28] Hurry V. M. Strand A. Tobiaeson M. Gardestrom P. Oquist G. (1995). Cold hardening of spring and winter wheat and rape results in differential effects on growth, carbon metabolism, and carbohydrate content. Plant Physiol. 109, 697–706. 10.1104/pp.109.2.697, PMID: 12228623 PMC157638

[ref29] Ivanov A. G. Morgan R. M. Gray G. R. Velitchkova M. Y. Huner N. P. A. (1998). Temperature/light dependent development of selective resistance to photoinhibition of photosystem I. FEBS Lett. 430, 288–292. 10.1016/S0014-5793(98)00681-4, PMID: 9688557

[ref30] Klughammer C. Schreiber U. (1994). An improved method, using saturating light pulses, for the determination of photosystem I quantum yield via P700^+^-absorbance changes at 830 nm. Planta 192, 261–268. 10.1007/BF00194461

[ref31] Kok B. Gassner E. S. Rurainski H. J. (1965). Photoinhibition of chloroplast reactions. Photochem. Photobiol. 4, 215–227. 10.1111/j.1751-1097.1965.tb05739.x, PMID: 5909993

[ref32] Kono M. Noguchi K. Terashima I. (2014). Roles of the cyclic electron flow around PSI (CEF-PSI) and O_2_-dependent alternative pathways in regulation of the photosynthetic electron flow in short-term fluctuating light in *Arabidopsis thaliana*. Plant Cell Physiol. 55, 990–1004. 10.1093/pcp/pcu033, PMID: 24553846

[ref33] Kouřil R. Wientjes E. Bultema J. B. Croce R. Boekema E. J. (2013). High-light vs. low-light: effect of light acclimation on photosystem II composition and organization in *Arabidopsis thaliana*. Biochim. Biophys. Acta Bioenerg. 1827, 411–419. 10.1016/j.bbabio.2012.12.003, PMID: 23274453

[ref34] Liu J. Last R. L. (2017). A chloroplast thylakoid lumen protein is required for proper photosynthetic acclimation of plants under fluctuating light environments. Proc. Natl. Acad. Sci. USA 114, E8110–E8117. 10.1073/pnas.1712206114, PMID: 28874535 PMC5617312

[ref35] Makino A. Sage R. F. (2007). Temperature response of photosynthesis in transgenic rice transformed with ‘sense’ or ‘antisense’ *rbcS*. Plant Cell Physiol. 48, 1472–1483. 10.1093/pcp/pcm118, PMID: 17804480

[ref36] Miyake C. Horiguchi S. Makino A. Shinzaki Y. Yamamoto H. Tomizawa K. I. (2005). Effects of light intensity on cyclic electron flow around PSI and its relationship to non-photochemical quenching of Chl fluorescence in tobacco leaves. Plant Cell Physiol. 46, 1819–1830. 10.1093/pcp/pci197, PMID: 16143595

[ref37] Miyata K. Ikeda H. Nakaji M. Kanel D. R. Terashima I. (2015). Rate constants of PSII photoinhibition and its repair, and PSII fluorescence parameters in field plants in relation to their growth light environments. Plant Cell Physiol. 56, 1841–1854. 10.1093/pcp/pcv107, PMID: 26203120

[ref38] Morosinotto T. Breton J. Bassi R. Croce R. (2003). The nature of a chlorophyll ligand in Lhca proteins determines the far red fluorescence emission typical of photosystem I. J. Biol. Chem. 278, 49223–49229. 10.1074/jbc.M309203200, PMID: 14504274

[ref39] Müller P. Li X. P. Niyogi K. K. (2001). Non-photochemical quenching. A response to excess light energy. Plant Physiol. 125, 1558–1566. 10.1104/pp.125.4.1558, PMID: 11299337 PMC1539381

[ref40] Munekage Y. Hojo M. Meurer J. Endo T. Tasaka M. Shikanai T. (2002). PGR5 is involved in cyclic electron flow around photosystem I and is essential for photoprotection in *Arabidopsis*. Cell 110, 361–371. 10.1016/S0092-8674(02)00867-X, PMID: 12176323

[ref41] Nishiyama Y. Allakhverdiev S. I. Murata N. (2011). Protein synthesis is the primary target of reactive oxygen species in the photoinhibition of photosystem II. Physiol. Plant. 142, 35–46. 10.1111/j.1399-3054.2011.01457.x, PMID: 21320129

[ref42] Noguchi K. Yamori W. Hikosaka K. Terashima I. (2015). Homeostasis of the temperature sensitivity of respiration over a range of growth temperatures indicated by a modified Arrhenius model. New Phytol. 207, 34–42. 10.1111/nph.13339, PMID: 25704334

[ref43] Oguchi R. Hikosaka K. Hirose T. (2003). Does the photosynthetic light-acclimation need change in leaf anatomy? Plant Cell Environ. 26, 505–512. 10.1046/j.1365-3040.2003.00981.x

[ref44] Pocock T. H. Hurry V. Savitch L. V. Huner N. (2001). Susceptibility to low-temperature photoinhibition and the acquisition of freezing tolerance in winter and spring wheat: the role of growth temperature and irradiance. Physiol. Plant. 113, 499–506. 10.1034/j.1399-3054.2001.1130408.x

[ref45] Porra R. J. Thompson W. A. Kriedemann P. E. (1989). Determination of accurate extinction coefficients and simultaneous equations for assaying chlorophylls *a* and *b* extracted with four different solvents: verification of the concentration of chlorophyll standards by atomic absorption spectroscopy. Biochim. Biophys. Acta Bioenerg. 975, 384–394. 10.1016/S0005-2728(89)80347-0

[ref46] Rajagopal S. Joly D. Gauthier A. Beauregard M. Carpentier R. (2005). Protective effect of active oxygen scavengers on protein degradation and photochemical function in photosystem I submembrane fractions during light stress. FEBS J. 272, 892–902. 10.1111/j.1742-4658.2004.04512.x, PMID: 15691324

[ref47] Roach T. Krieger-Liszkay A. (2014). Regulation of photosynthetic electron transport and photoinhibition. Curr. Protein Pept. Sci. 15, 351–362. 10.2174/1389203715666140327105143, PMID: 24678670 PMC4030316

[ref48] Sage R. F. Way D. A. Kubien D. S. (2008). Rubisco, Rubisco activase, and global climate change. J. Exp. Bot. 59, 1581–1595. 10.1093/jxb/ern053, PMID: 18436544

[ref49] Savitch L. V. Barker-Åstrom J. Ivanov A. G. Hurry V. Öquist G. Huner N. P.. (2001). Cold acclimation of *Arabidopsis thaliana* results in incomplete recovery of photosynthetic capacity, associated with an increased reduction of the chloroplast stroma. Planta 214, 295–303. 10.1007/s004250100622, PMID: 11800395

[ref50] Savitch L. V. Harney T. Huner N. (2000). Sucrose metabolism in spring and winter wheat in response to high irradiance, cold stress and cold acclimation. Physiol. Plant. 108, 270–278. 10.1034/j.1399-3054.2000.108003270.x

[ref51] Scheller H. V. Haldrup A. (2005). Photoinhibition of photosystem I. Planta 221, 5–8. 10.1007/s00425-005-1507-7, PMID: 15782347

[ref52] Schumann T. Paul S. Melzer M. Dörmann P. Jahns P. (2017). Plant growth under natural light conditions provides highly flexible short-term acclimation properties toward high light stress. Front. Plant Sci. 8:681. 10.3389/fpls.2017.00681, PMID: 28515734 PMC5413563

[ref53] Sejima T. Takagi D. Fukayama H. Makino A. Miyake C. (2014). Repetitive short-pulse light mainly inactivates photosystem I in sunflower leaves. Plant Cell Physiol. 55, 1184–1193. 10.1093/pcp/pcu061, PMID: 24793753

[ref54] Shaku K. Shimakawa G. Hashiguchi M. Miyake C. (2015). Reduction-induced suppression of electron flow (RISE) in the photosynthetic electron transport system of *Synechococcus elongatus* PCC 7942. Plant Cell Physiol. 57, 1443–1453. 10.1093/pcp/pcv198 26707729

[ref55] Shimakawa G. Ishizaki K. Tsukamoto S. Tanaka M. Sejima T. Miyake C. (2017). The liverwort, *Marchantia*, drives alternative electron flow using a flavodiiron protein to protect PSI. Plant Physiol. 173, 1636–1647. 10.1104/pp.16.01038, PMID: 28153920 PMC5338653

[ref56] Shimakawa G. Murakami A. Niwa K. Matsuda Y. Wada A. Miyake C. (2019). Comparative analysis of strategies to prepare electron sinks in aquatic photoautotrophs. Photosynth. Res. 139, 401–411. 10.1007/s11120-018-0522-z 29845382

[ref57] Shimakawa G. Shaku K. Miyake C. (2016). Oxidation of P700 in photosystem I is essential for the growth of cyanobacteria. Plant Physiol. 172, 1443–1450. 10.1104/pp.16.01227, PMID: 27613853 PMC5100761

[ref58] Sonoike K. (1995). Selective photoinhibition of photosystem I in isolated thylakoid membranes from cucumber and spinach. Plant Cell Physiol. 36, 825–830. 10.1093/oxfordjournals.pcp.a078827

[ref59] Sonoike K. (1996a). Photoinhibition of photosystem I: its physiological significance in the chilling sensitivity of plants. Plant Cell Physiol. 37, 239–247. 10.1093/oxfordjournals.pcp.a028938

[ref60] Sonoike K. (1996b). Degradation of *psaB* gene product, the reaction center subunit of photosystem I, is caused during photoinhibition of photosystem I: possible involvement of active oxygen species. Plant Sci. 115, 157–164. 10.1016/0168-9452(96)04341-5

[ref61] Sonoike K. (1998). Various aspects of inhibition of photosynthesis under light/chilling stress: “photoinhibition at chilling temperatures” versus “chilling damage in the light”. J. Plant Res. 111, 121–129. 10.1007/BF02507158

[ref62] Sonoike K. (1999). The different roles of chilling temperatures in the photoinhibition of photosystem I and photosystem II. J. Photochem. Photobiol. B Biol. 48, 136–141. 10.1016/S1011-1344(99)00030-5

[ref500] Sonoike K. (2011). Photoinhibition of photosystem I. Physiol. Plant. 142, 56–64. 10.1111/j.1399-3054.2010.01437.x 21128947

[ref63] Sonoike K. Ishibashi M. Watanabe A. (1995a). “Chilling sensitive steps in leaves of *Phaseolus vulgaris* L. examination of the effects of growth irradiances on PSI photoinhibition” in Photosynthesis: From light to biosphere. ed. Matis P., vol. IV (The Hague: Kluwer Academic Publishers), 853–856.

[ref64] Sonoike K. Terashima I. (1994). Mechanism of photosystem-I photoinhibition in leaves of *Cucumis sativus* L. Planta 194, 287–293. 10.1007/BF01101690

[ref65] Sonoike K. Terashima I. Iwaki M. Itoh S. (1995b). Destruction of photosystem I iron-sulfur centers in leaves of *Cucumis sativus* L. by weak illumination at chilling temperatures. FEBS Lett. 362, 235–238. 10.1016/0014-5793(95)00254-7 7720879

[ref66] Suorsa M. Järvi S. Grieco M. Nurmi M. Pietrzykowska M. Rantala M.. (2012). PROTON GRADIENT REGULATION5 is essential for proper acclimation of *Arabidopsis* photosystem I to naturally and artificially fluctuating light conditions. Plant Cell 24, 2934–2948. 10.1105/tpc.112.097162, PMID: 22822205 PMC3426124

[ref67] Suorsa M. Rantala M. Mamedov F. Lespinasse M. Trotta A. Grieco M.. (2015). Light acclimation involves dynamic re-organization of the pigment-protein megacomplexes in non-appressed thylakoid domains. Plant J. 84, 360–373. 10.1111/tpj.13004, PMID: 26332430

[ref68] Takagi D. Amako K. Hashiguchi M. Fukaki H. Ishizaki K. Goh T. (2017b). Chloroplastic ATP synthase builds up a *proton motive force* preventing production of reactive oxygen species in photosystem I. Plant J. 91, 306–324. 10.1111/tpj.13566 28380278

[ref69] Takagi D. Hashiguchi M. Sejima T. Makino A. Miyake C. (2016b). Photorespiration provides the chance of cyclic electron flow to operate for the redox-regulation of P700 in photosynthetic electron transport system of sunflower leaves. Photosynth. Res. 129, 279–290. 10.1007/s11120-016-0267-5 27116126

[ref70] Takagi D. Ishizaki K. Hanawa H. Mabuchi T. Shimakawa G. Yamamoto H. (2017a). Diversity of strategies for escaping reactive oxygen species production within photosystem I among land plants: P700 oxidation system is prerequisite for alleviating photoinhibition in photosystem I. Physiol. Plant. 161, 56–74. 10.1111/ppl.12562 28295410

[ref71] Takagi D. Miyake C. (2018). Proton gradient regulation 5 supports linear electron flow to oxidize photosystem I. Physiol. Plant. 164, 337–348. 10.1111/ppl.12723, PMID: 29604096

[ref72] Takagi D. Takumi S. Hashiguchi M. Sejima T. Miyake C. (2016a). Superoxide and singlet oxygen produced within the thylakoid membranes both cause photosystem I photoinhibition. Plant Physiol. 171, 1626–1634. 10.1104/pp.16.00246 26936894 PMC4936555

[ref73] Telfer A. Dhami S. Bishop S. M. Phillips D. Barber J. (1994). β-carotene quenches singlet oxygen formed by isolated photosystem II reaction centers. Biochemistry 33, 14469–14474. 10.1021/bi00252a013 7981207

[ref74] Terao T. Katoh S. (1996). Antenna sizes of photosystem I and photosystem II in chlorophyll *b*-deficient mutants of rice. Evidence for an antenna function of photosystem II centers that are inactive in electron transport. *Plant Cell Physiol*. 37, 307–312. 10.1093/oxfordjournals.pcp.a028947

[ref75] Terashima I. Funayama S. Sonoike K. (1994). The site of photoinhibition in leaves of *Cucumis sativus* L. at low temperatures is photosystem I, not photosystem II. Planta 193, 300–306. 10.1007/BF00192544

[ref76] Terashima I. Kashino Y. Katoh S. (1991a). Exposure of leaves of *Cucumis sativus* L. to low temperatures in the light causes uncoupling of thylakoids I. Studies with isolated thylakoids. Plant Cell Physiol. 32, 1267–1274. 10.1093/oxfordjournals.pcp.a078205

[ref77] Terashima I. Sonoike K. Kawazu T. Katoh S. (1991b). Exposure of leaves of *Cucumis sativus* L. to low temperatures in the light causes uncoupling of thylakoids II. Non-destructive measurements with intact leaves. Plant Cell Physiol. 32, 1275–1283. 10.1093/oxfordjournals.pcp.a078206

[ref78] Tikkanen M. Grieco M. Kangasjärvi S. Aro E. M. (2010). Thylakoid protein phosphorylation in higher plant chloroplasts optimizes electron transfer under fluctuating light. Plant Physiol. 152, 723–735. 10.1104/pp.109.150250, PMID: 19965965 PMC2815896

[ref79] Tikkanen M. Grieco M. Nurmi M. Rantala M. Suorsa M. Aro E. M. (2012). Regulation of the photosynthetic apparatus under fluctuating growth light. Philos. Trans. R. Soc. Lond. B Biol. Sci. 367, 3486–3493. 10.1098/rstb.2012.0067 23148275 PMC3497072

[ref80] Tikkanen M. Mekala N. R. Aro E. M. (2014). Photosystem II photoinhibition-repair cycle protects Photosystem I from irreversible damage. Biochim. Biophys. Acta Bioenerg. 1837, 210–215. 10.1016/j.bbabio.2013.10.001 24161359

[ref81] Tiwari A. Mamedov F. Grieco M. Suorsa M. Jajoo A. Styring S.. (2016). Photodamage of iron–sulphur clusters in photosystem I induces non-photochemical energy dissipation. Nat. Plants 2:16035. 10.1038/nplants.2016.35, PMID: 27249566

[ref82] Tjus S. E. Møller B. L. Scheller H. V. (1999). Photoinhibition of photosystem I damages both reaction centre proteins PSI-A and PSI-B and acceptor-side located small photosystem I polypeptides. Photosynth. Res. 60, 75–86. 10.1023/A:1006283618695

[ref83] Wada S. Suzuki Y. Takagi D. Miyake C. Makino A. (2018). Effects of genetic manipulation of the activity of photorespiration on the redox state of photosystem I and its robustness against excess light stress under CO_2_-limited conditions in rice. Photosynth. Res. 137, 431–441. 10.1007/s11120-018-0515-y, PMID: 29761327

[ref84] Walters R. G. (2005). Towards an understanding of photosynthetic acclimation. J. Exp. Bot. 56, 435–447. 10.1093/jxb/eri060, PMID: 15642715

[ref85] Walters R. G. Shephard F. Rogers J. J. Rolfe S. A. Horton P. (2003). Identification of mutants of *Arabidopsis* defective in acclimation of photosynthesis to the light environment. Plant Physiol. 131, 472–481. 10.1104/pp.015479, PMID: 12586872 PMC166824

[ref86] Ware M. A. Belgio E. Ruban A. V. (2015). Photoprotective capacity of non-photochemical quenching in plants acclimated to different light intensities. Photosynth. Res. 126, 261–274. 10.1007/s11120-015-0102-4, PMID: 25702085

[ref87] Wientjes E. Oostergetel G. T. Jansson S. Boekema E. J. Croce R. (2009). The role of Lhca complexes in the supramolecular organization of higher plant photosystem I. J. Biol. Chem. 284, 7803–7810. 10.1074/jbc.M808395200, PMID: 19139095 PMC2658074

[ref88] Yamatani H. Kohzuma K. Nakano M. Takami T. Kato Y. Hayashi Y.. (2018). Impairment of Lhca4, a subunit of LHCI, causes high accumulation of chlorophyll and the stay-green phenotype in rice. J. Exp. Bot. 69, 1027–1035. 10.1093/jxb/erx468, PMID: 29304198 PMC6019047

[ref89] Yamori W. Hikosaka K. Way D. A. (2014). Temperature response of photosynthesis in C3, C4, and CAM plants: temperature acclimation and temperature adaptation. *Photosynth. Res*. 119, 101–117. 10.1007/s11120-013-9874-6, PMID: 23801171

[ref90] Zivcak M. Brestic M. Balatova Z. Drevenakova P. Olsovska K. Kalaji H. M.. (2013). Photosynthetic electron transport and specific photoprotective responses in wheat leaves under drought stress. Photosynth. Res. 117, 529–546. 10.1007/s11120-013-9885-3, PMID: 23860828

[ref91] Zivcak M. Brestic M. Kunderlikova K. Sytar O. Allakhverdiev S. I. (2015). Repetitive light pulse-induced photoinhibition of photosystem I severely affects CO_2_ assimilation and photoprotection in wheat leaves. *Photosynth. Res*. 126, 449–463. 10.1007/s11120-015-0121-1, PMID: 25829027

