# Supplementary material for: Growth Light Environment Changes the Sensitivity of Photosystem I Photoinhibition Depending on Common Wheat Cultivars
Source: Front Plant Sci. 2019 Jun 4;10:686. doi: 10.3389/fpls.2019.00686 (PMC6557977; doi:10.3389/fpls.2019.00686)
Supplement: Supplementary file 1 [file Data_Sheet_1.pdf]

Title: Growth light environment changes the sensitivity of photosystem I photoinhibition by depending on common wheat cultivar

Running title: Cultivar differences in PSI photoinhibition

Authors

Daisuke Takagi[1,2], Hiroaki Ihara[1], Shigeo Takumi[1], Chikahiro Miyake[1,2*]

Author's address

[1]Department of Biological and Environmental Science, Graduate School of Agricultural Science, Kobe University, 1-1 Rokkodai, Nada, Kobe, 657-8501 Japan

[2]Core Research for Environmental Science and Technology, Japan Science and Technology Agency, 7 Gobancho, Chiyoda-ku, Tokyo 102-0076, Japan

[*]**For correspondence**

Corresponding author: Chikahiro Miyake

(fax; +81-78-803-5851, cmiyake@hawk.kobe.u-ac.jp)

Keywords

Photosystem I, Photoinhibition, Reactive oxygen species, Light acclimation, Wheat (*Triticum aestivum* L.)

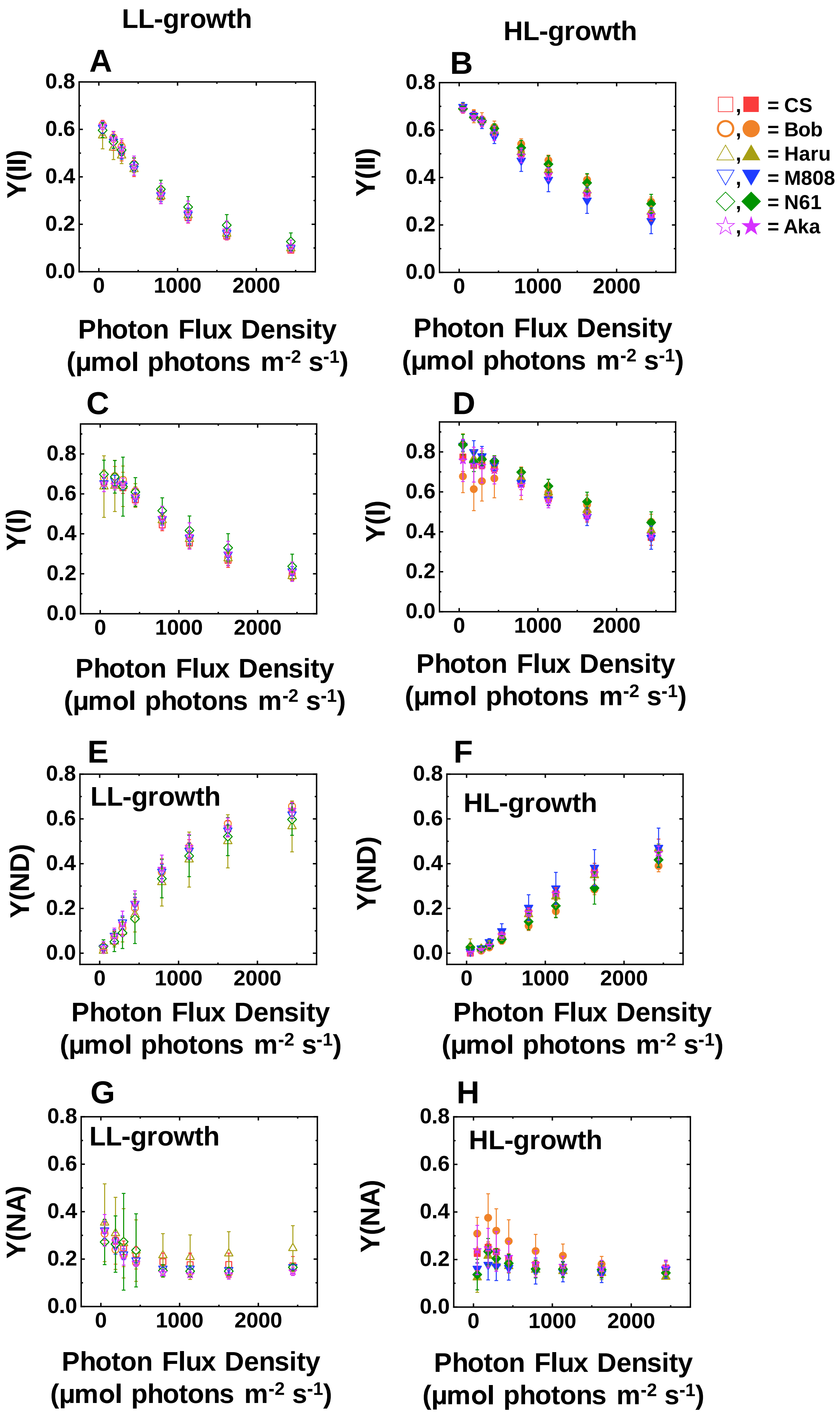

LL-growth
HL-growth
A
B
C
D
E
F
G
H
Y(II)
Y(I)
Y(ND)
Y(NA)
0.0
0.2
0.4
0.6
0.8
0
1000
2000
Photon Flux Density
(μmol photons m-2 s-1)
LL-growth
HL-growth
= CS
= Bob
= Haru
= M808
= N61
= Aka

Supplemental Figure S1

The study of the cultivar differences in Y(II), Y(I), Y(ND) and Y(NA) among wheat plants grown under LL- and HL-conditions respectively. The data were same as shown in Figures 2-5. Data are expressed as mean ± SD of three to four independent experiments. To examine the significant differences among the wheat cultivars, analysis of variance (ANOVA) and Tukey-Kramer honestly significant difference (HSD) test were conducted at the maximum light intensity. Red squares indicate the results of CS, orange circles indicate the results of Bob, dark yellow triangles indicate the results of Haru, blue inverted-triangles indicate the results of M808, green diamonds indicate the results of N61, and purple stars indicate the results of Aka. Open symbols indicate the results of LL-grown wheat plants, and closed symbols indicate the result of HL-grown wheat plants.

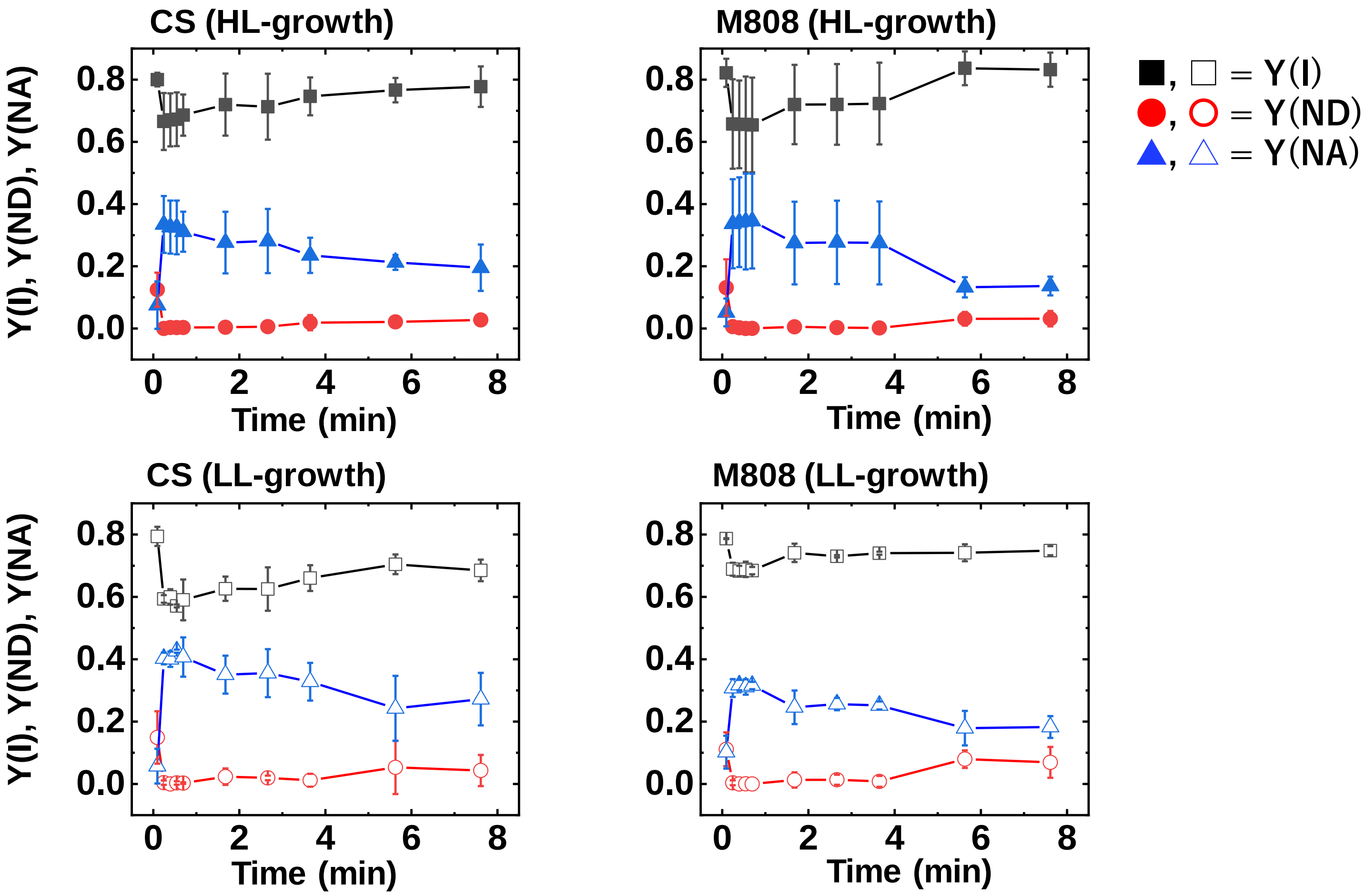

Supplemental Figure S2

Analysis of the rapid change in Y(I), Y(ND) and Y(NA) at the beginning of rSP illumination in CS and M808 grown under both LL- (open symbols) and HL-conditions (closed symbols). Before rSP illumination, the wheat plants were adapted in the dark for at least 1 h. In these experiments, the wheat plants are illuminated with a short pulse (300 ms, 20,000 μmol photons m^{-2} s^{-1}) with the different illuminating intervals. From the time 0 min to 1 min, the short pulse was illuminated every 10 s. From 1 min to 4 min, the short pulse was illuminated every 1 min. From 4 min to 8 min, the short pulse was illuminated every 2 min. Data are expressed as mean ± SD of three independent experiments. Black squares indicate Y(I), red circles indicate Y(NA) and blue triangles indicate Y(NA).

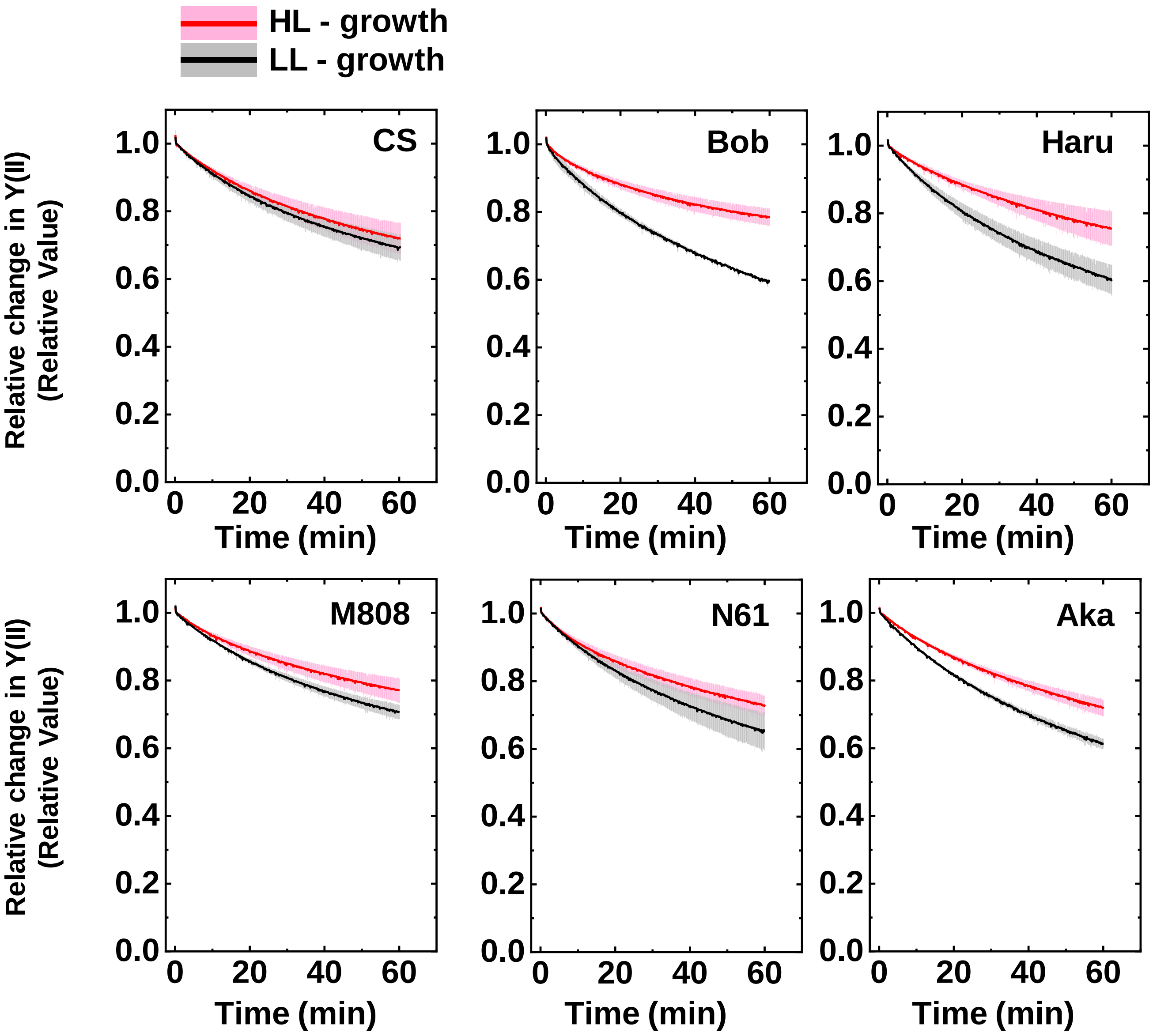

Supplement Figure S3

The susceptibility of PSII photoinhibition to rSP illumination in wheat cultivars grown under LL- and HL-conditions. (A) showed the time-course analysis of the relative change in Y(II) during rSP illumination under atmospheric conditions (21 kPa O_2, 40 Pa CO_2) at 25°C. Before rSP illumination, wheat plants were adapted in the dark for at least 1 h. Wheat leaves were illuminated every 10 s with a saturating pulse (300 ms, 20,000 μE m^{-2} s^{-1}) for 1 h in the dark. Data were expressed as mean ± SD of three to five independent experiments. Black line indicates the mean result of LL-grown wheat cultivars and gray shadow indicates the SD of the results of the LL-grown wheat cultivars. Red line indicates the mean results of the LL-grown wheat cultivars and pink shadow indicates the SD of the results of the HL-grown wheat cultivars.

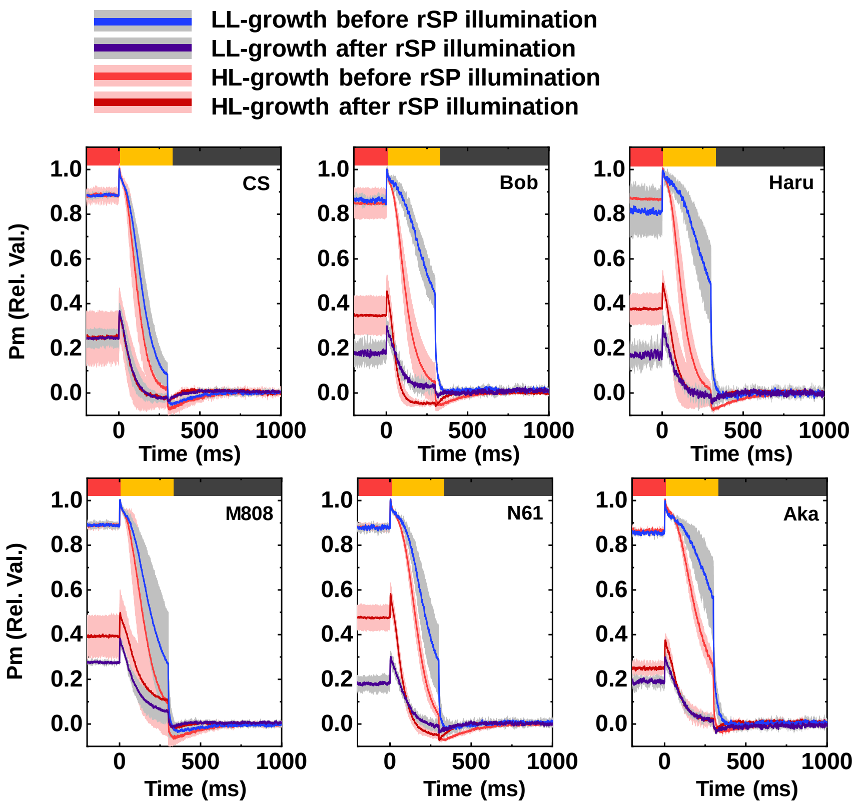

Supplemental Figure S4

The P700$^+$ kinetics in wheat cultivars under the determination of Pm before and after rSP illumination. Before the determination of Pm, wheat plants were adapted in the dark for 30 min. To determine Pm value, Far-red (FR) light was illuminated to wheat leaves, and steady-state P700$^+$ value was obtained. Subsequently, pulse illumination (300 ms, 20,000 µmol photons m^{-2} s^{-1}) was applied and determined maximum P700$^+$ signals by using extrapolation method (see Materials and Methods). After pulse illumination, stable zero level of P700$^+$ was determined at the steady state. Data were expressed as mean ± SD of three to five independent experiments. P700$^+$ signal was normalized by the Pm before rSP illumination in LL- and HL-grown wheat plants respectively. Blue lines with gray shadows indicate the results of LL-grown wheat plants before rSP illumination. Dark blue with gray shadows indicates the results of LL-grown wheat plants after rSP illumination. Red lines with pink shadows indicate the results of HL-grown wheat plants before rSP illumination. Dark red lines with pink shadows indicates the results of LL-grown wheat plants after rSP illumination. The red bars indicate the FR-illumination, yellow bars indicate the pulse illumination, and dark gray bars indicate the dark respectively.

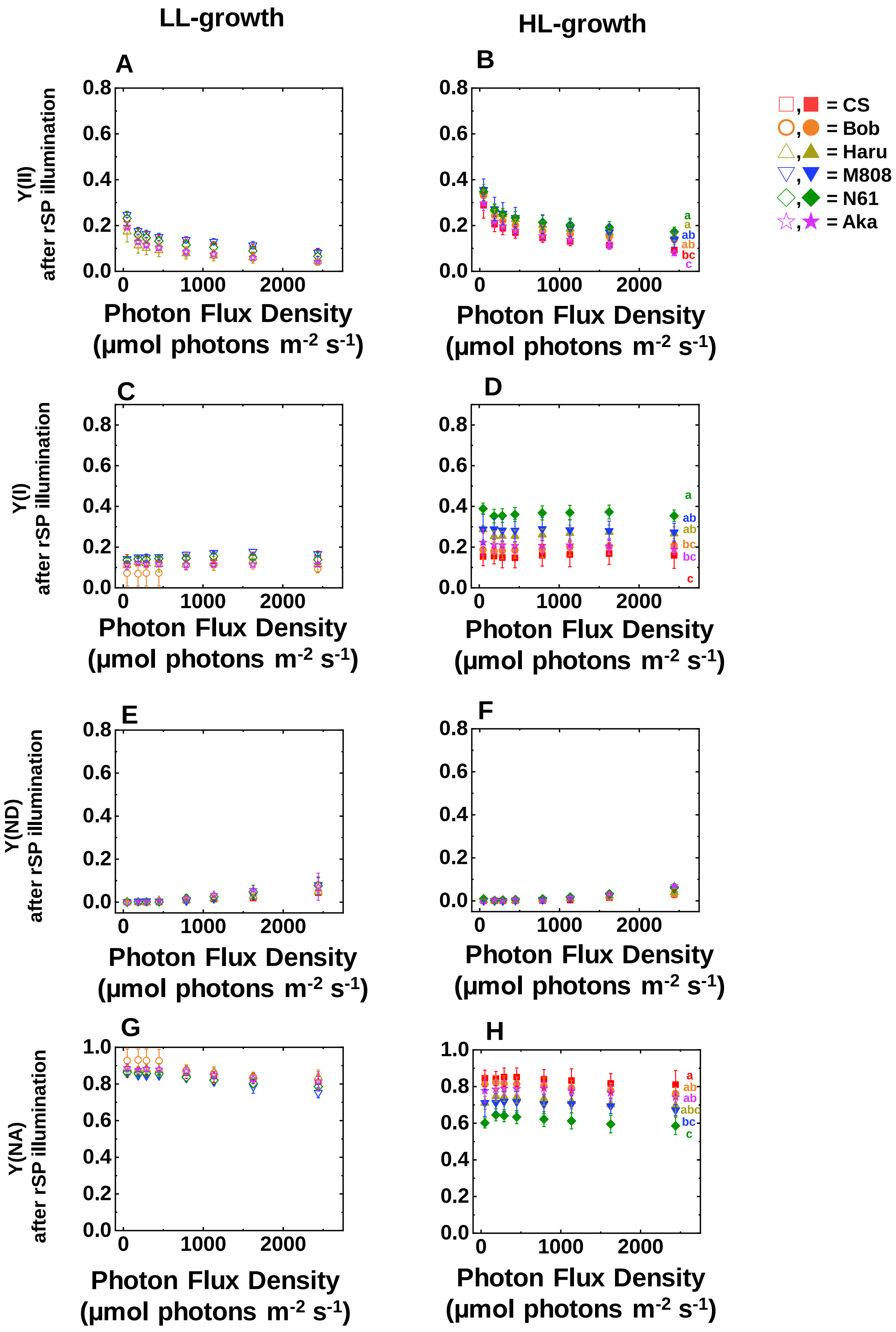

LL-growth
HL-growth
A
B
C
D
E
F
G
H
Y(II) after rSP illumination
Y(I) after rSP illumination
Y(ND) after rSP illumination
Y(NA) after rSP illumination
Photon Flux Density (µmol photons m-2 s-1)
□,■ = CS
○,● = Bob
△,▲ = Haru
▽,▼ = M808
◇,◆ = N61
☆,★ = Aka

Supplemental Figure S5

The study of the cultivar differences in Y(II), Y(I), Y(ND) and Y(NA) among wheat plants grown under LL- and HL-conditions after rSP illumination respectively. The data were same as shown in Figures 7-10. Data are expressed as mean ± SD of three to four independent experiments. To examine the significant differences among the wheat cultivars, analysis of variance (ANOVA) and Tukey-Kramer honestly significant difference (HSD) test were conducted at the maximum light intensity. Different letters adjacent to the symbols indicate a significant difference between those wheat cultivars (Tukey-Kramer HSD test, $p < 0.05$). Red squares indicate the results of CS, orange circles indicate the results of Bob, dark yellow triangles indicate the results of Haru, blue inverted-triangles indicate the results of M808, green diamonds indicate the results of N61, and purple stars indicate the results of Aka. Open symbols indicate the results of LL-grown wheat plants, and closed symbols indicate the result of HL-grown wheat plants.

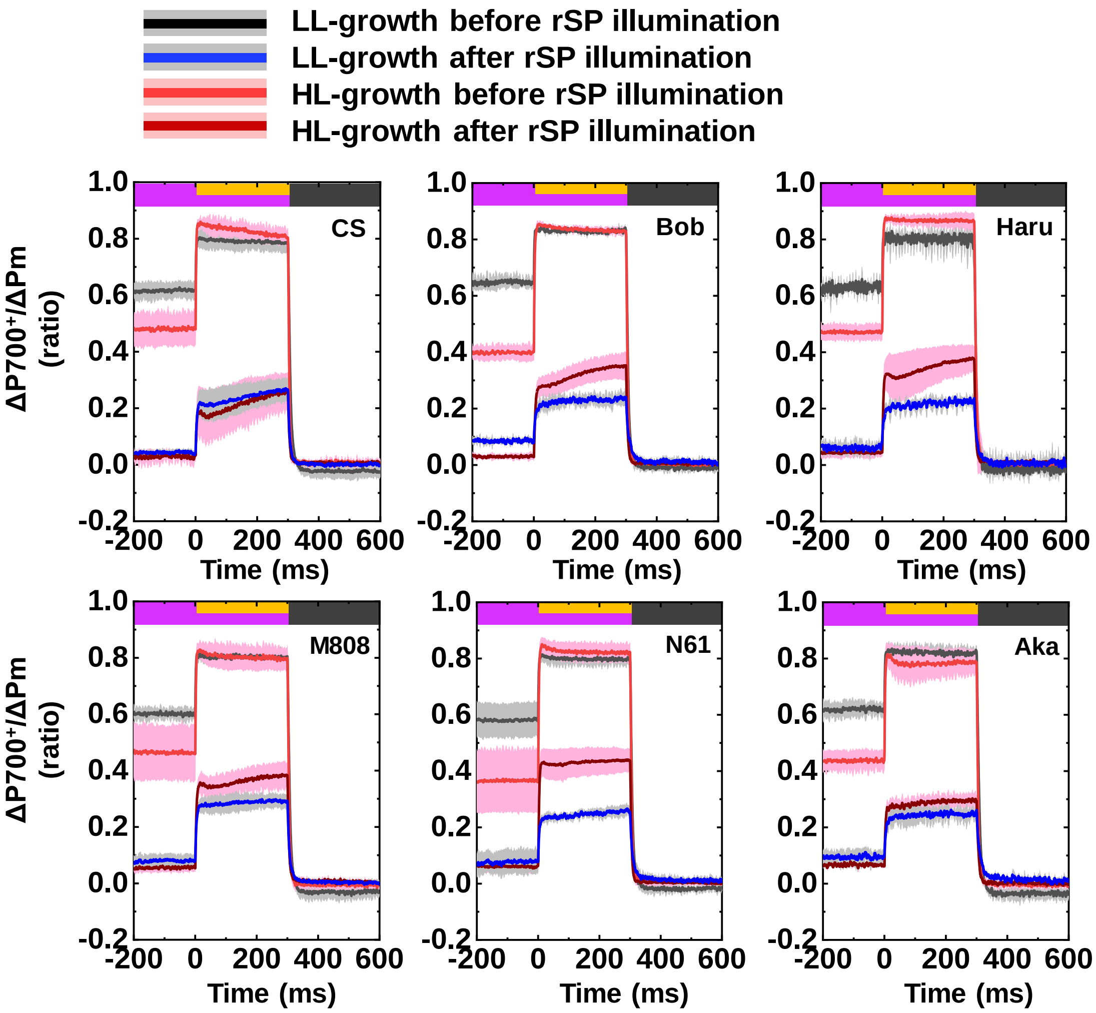

Supplemental Figure S6

The $P700^+$ kinetics in wheat cultivars under the determination of Y(I), Y(ND) and Y(NA) before and after rSP illumination. To determine the Y(I), Y(ND) and Y(NA), AL (2439 μmol photons m^{-2} s^{-1}) was illuminated. Subsequently, at the steady-state conditions, pulse illumination (300 ms, 20,000 μmol photons m^{-2} s^{-1}) was applied and determined maximum $P700^+$ signals by using extrapolation method (see Materials and Methods). After pulse illumination, stable zero level of $P700^+$ was determined at the steady state. Data were expressed as mean ± SD of three to five independent experiments. $P700^+$ signal was normalized by the Pm before rSP illumination in LL- and HL-grown wheat plants respectively. Black lines with gray shadows indicate the results of LL-grown wheat plants before rSP illumination. Blue with gray shadows indicates the results of LL-grown wheat plants after rSP illumination. Red lines with pink shadows indicate the results of HL-grown wheat plants before rSP illumination. Dark red lines with pink shadows indicates the results of LL-grown wheat plants after rSP illumination. The purple bars indicate the AL-illumination, yellow bars indicate the pulse illumination, and dark gray bars indicate the dark respectively.

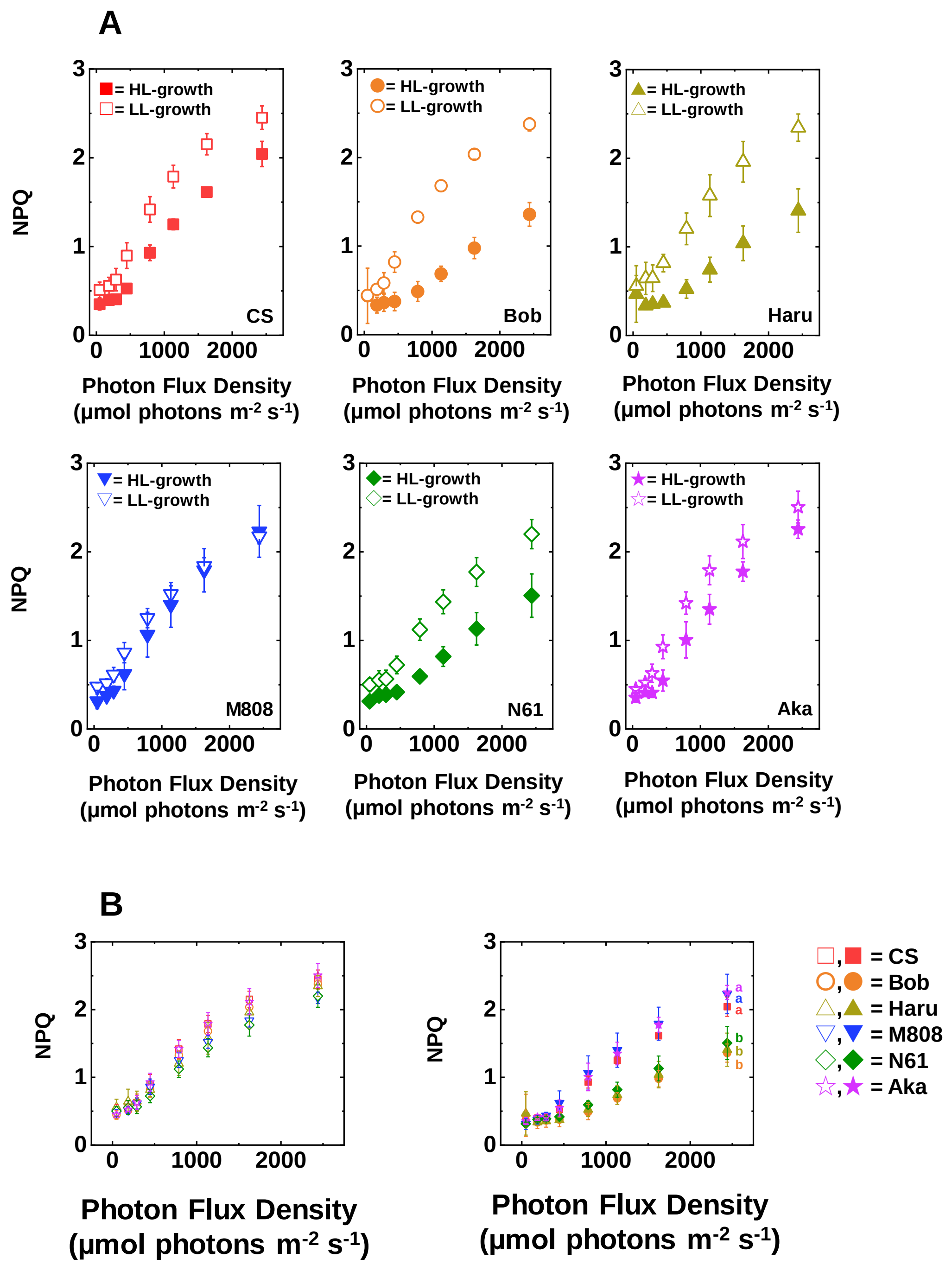

Supplemental Figure S7

The response of NPQ to light intensity in six wheat cultivars grown under LL (open symbol) and HL (closed symbol) conditions. Figure (A) displays the difference in NPQ between wheat plants grown under LL- and HL-conditions in respective wheat cultivars. Figure (B) displays the cultivar differences grown under LL- and HL-conditions respectively. The data of (A) and (B) is same. The measurement was conducted under atmospheric conditions (21 kPa O_2, 40 Pa CO_2) at 25°C. Data were expressed as mean ± SD of three to five independent experiments. Red squares indicate the results of CS, orange circles indicate the results of Bob, dark yellow triangles indicate the results of Haru, blue inverted-triangles indicate the results of M808, green diamonds indicate the results of N61, and purple stars indicate the results of Aka

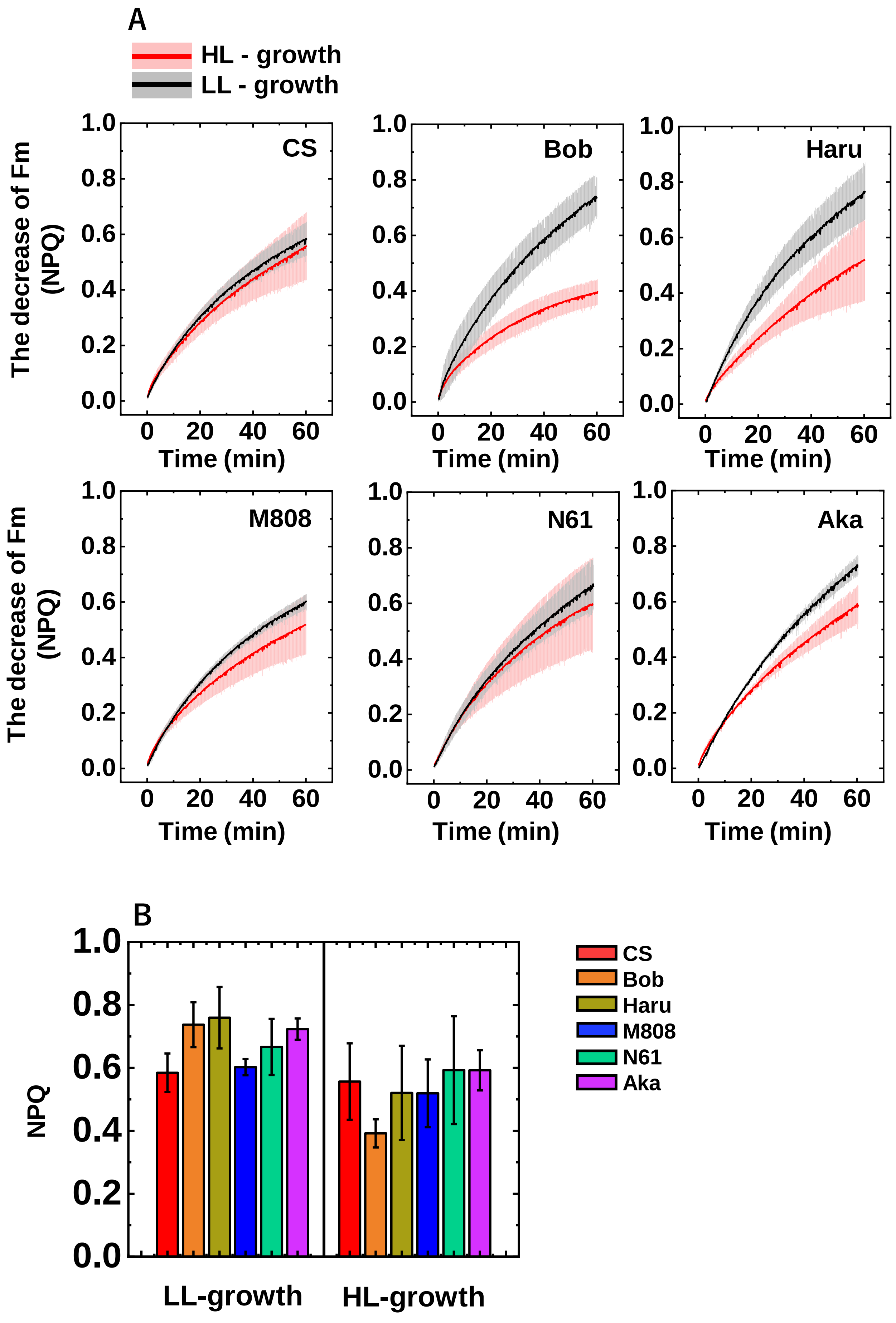

A
HL - growth
LL - growth
The decrease of Fm (NPQ)
CS
Bob
Haru
M808
N61
Aka
Time (min)
1.0
0.8
0.6
0.4
0.2
0.0
0
20
40
60
B
NPQ
CS
Bob
Haru
M808
N61
Aka
LL-growth
HL-growth

Supplemental Figure S8

The change in the decrease of Fm (NPQ) during rSP illumination in wheat cultivars grown under LL- and HL-conditions. (A) showed the time-course analysis of the decrease of Fm (NPQ) during rSP illumination under atmospheric conditions (21 kPa O_2, 40 Pa CO_2) at 25°C. Before rSP illumination, wheat plants were adapted in the dark for at least 1 h. Wheat leaves were illuminated every 10 s with a saturating pulse (300 ms, 20,000 μE m^{-2} s^{-1}) for 1 h in the dark. Data were expressed as mean ± SD of three to five independent experiments. Black line indicates the mean result of LL-grown wheat cultivars and gray shadow indicates the SD of the results of the LL-grown wheat cultivars. Red line indicates the mean results of the LL-grown wheat cultivars and pink shadow indicates the SD of the results of the HL-grown wheat cultivars. The final values of NPQ during rSP illumination in wheat cultivars grown under LL- and HL-conditions are shown in (B). Data were expressed as mean ± SD of three to five independent experiments. Red bars indicate the results of CS, orange bars indicate the results of Bob, dark yellow bars indicate the results of Haru, blue bars indicate the results of M808, green bars indicate the results of N61, and purple bars indicate the results of Aka.
